# *Bradyrhizobium agreste* sp. nov., *Bradyrhizobium glycinis* sp. nov. and *Bradyrhizobium diversitatis* sp. nov., isolated from a biodiversity hotspot of the genus *Glycine* in Western Australia

**DOI:** 10.1099/ijsem.0.004742

**Published:** 2021-03-12

**Authors:** Milena Serenato Klepa, Luisa Caroline Ferraz Helene, Graham O’Hara, Mariangela Hungria

**Affiliations:** ^1^​ Embrapa Soja, C.P. 231, 86001-970, Londrina, Paraná, Brazil; ^2^​ Coordenação de Aperfeiçoamento de Pessoal de Nível Superior, SBN, Quadra 2, Bloco L, Lote 06, Edifício Capes, 70.040-020, Brasília, Distrito Federal, Brazil; ^3^​ Department of Microbiology, Universidade Estadual de Londrina, C.P. 10011, 86057-970, Londrina, Paraná, Brazil; ^4^​ Centre for Rhizobium Studies (CRS), Murdoch University 90 South St. Murdoch, WA, Australia

**Keywords:** *Bradyrhizobium*, wild soybean, *Glycine*, nodulation, MLSA, ANI, dDDH

## Abstract

Strains of the genus *Bradyrhizobium* associated with agronomically important crops such as soybean (*Glycine max*) are increasingly studied; however, information about symbionts of wild *Glycine* species is scarce. Australia is a genetic centre of wild *Glycine* species and we performed a polyphasic analysis of three *Bradyrhizobium* strains—CNPSo 4010^T^, CNPSo 4016^T^, and CNPSo 4019^T^—trapped from Western Australian soils with *Glycine clandestina*, *Glycine tabacina* and *Glycine max*, respectively. The phylogenetic tree of the 16S rRNA gene clustered all strains into the *Bradyrhizobium japonicum* superclade; strains CNPSo 4010^T^ and CNPSo 4016^T^ had *Bradyrhizobium yuanmingense* CCBAU 10071^T^ as the closest species, whereas strain CNPSo 4019^T^ was closer to *Bradyrhizobium liaoningense* LMG 18230^T^. The multilocus sequence analysis (MLSA) with five housekeeping genes—*dnaK*, *glnII*, *gyrB*, *recA* and *rpoB*—confirmed the same clusters as the 16S rRNA phylogeny, but indicated low similarity to described species, with nucleotide identities ranging from 93.6 to 97.6% of similarity. Considering the genomes of the three strains, the average nucleotide identity and digital DNA–DNA hybridization values were lower than 94.97 and 59.80 %, respectively, with the closest species. In the *nodC* phylogeny, strains CNPSo 4010^T^ and CNPSo 4019^T^ grouped with *Bradyrhizobium zhanjiangense* and *Bradyrhizobium ganzhouense*, respectively, while strain CNPSo 4016^T^ was positioned separately from the all symbiotic *Bradyrhizobium* species. Other genomic (BOX-PCR), phenotypic and symbiotic properties were evaluated and corroborated with the description of three new lineages of *Bradyrhizobium*. We propose the names of *Bradyrhizobium agreste* sp. nov. for CNPSo 4010^T^ (=WSM 4802^T^=LMG 31645^T^) isolated from *Glycine clandestina*, *Bradyrhizobium glycinis* sp. nov. for CNPSo 4016^T^ (=WSM 4801^T^=LMG 31649^T^) isolated from *Glycine tabacina* and *Bradyrhizobium diversitatis* sp. nov. for CNPSo 4019^T^ (=WSM 4799^T^=LMG 31650^T^) isolated from *G. max*.

## Introduction

Balanced levels of N in soil are required to obtain high productivities and healthy plants. Biological nitrogen fixation (BNF) is carried out by a restricted group of prokaryotes able to reduce the atmospheric dinitrogen (N_2_) to ammonium (NH_3_), the assimilable form by plants [1, 2]. The highest level of evolution of BNF occurs with the symbiotic interaction between diazotrophic bacteria collectively known as rhizobia and members of the family Fabaceae (=Leguminosae). The symbiosis involves a mutual exchange of molecular signals, culminating in the development of specialized structures on roots, and occasionally on stems, called nodules, in which the BNF process takes place [3, 4]. A variety of elite rhizobial strains have been introduced in agricultural systems for over a century as a sustainable way to improve soil fertility and crop yield, in addition to lowering costs, due to the replacement of chemical N-fertilizers [5, 6]. One remarkable example relies on the symbiosis of *Bradyrhizobium* with the soybean (*Glycine max*) crop in Brazil, saving billions of dollars every year [7, 8].

Several *Bradyrhizobium* species have been isolated from nodules of soybean, mostly grown in China, the main genetic centre of this legume species [9–11]. However, *Bradyrhizobium* is a geographically widespread genus that nodulates several legume tribes, from herbaceous to trees, mainly in tropical regions [12–15]. Recently, Helene *et al*. [16] reported a high diversity of *Bradyrhizobium* strains isolated from Western Australian soils using species of *Glycine* species as trapping hosts. It is worth mentioning that the genus *Glycine* is split into two subgenera: *Soja*, with *Glycine max* and *Glycine soja* species, and *Glycine*, which includes 25 species of wild soybean, most indigenous to Australia [17–20]; for this reason, although not often mentioned, Australia is a great hotspot of diversity of the genus *Glycine* [21].

Populations of Australian indigenous *Glycine* species are distributed all over the country and are the wild relatives of the economically important soybean crop [17, 21, 22]. Due to the inhospitable conditions of most areas growing indigenous *Glycine* in Australia, bioprospecting their genomes may help to understand and improve the adaptation of soybean to climate change [21]. In addition, to investigate the diversity of rhizobia symbionts of indigenous *Glycine* species in soils of Western Australia should provide valuable information for biotechnological applications. Here, we report a polyphasic study with three lineages of *Bradyrhizobium* isolated from *Glycine clandestina, G. tabacina* and *G. max* that resulted in the description of three new species.

## Isolation and ecology

Strains CNPSo 4010^T^, CNPSo 4016^T^ and CNPSo 4019^T^ were isolated from root nodules of three different species, *G. clandestina* JC Wendl [23], *G. tabacina* (Labill.) Benth [24] and *G. max*, respectively, used as trap plants in Western Australian soil, as previously reported by our group [16]. Information about the strains used in this study as well as the sampling soil points are shown in Table 1.

**Table 1.** Strains used in this study

Species/strain name	Other nomenclatures	Original host species	Geographical origin	Reference
*Bradyrhizobium agreste* CNPSo 4010^T^	WSM 4802^T^=LMG 31645	*Glycine clandestina*	Kununurra, Australia	Helene *et al*. [16]
*Bradyrhizobium glycinis* CNPSo 4016^T^	WSM 4801^T^=LMG 31649^T^	*Glycine tabacina*	Kununurra, Australia	Helene *et al*. [16]
*Bradyrhizobium diversitatis* CNPSo 4019^T^	WSM 4799^T^=LMG 31650^T^	*Glycine max*	Nambung, Australia	Helene *et al*. [16]
*Bradyrhizobium fredereickii* CNPSo 3426^T^	USDA 10052^T^=U686^T^=CL 20^T^	*Chamaecrista fasciculata*	Missouri, USA	Urquiaga *et al*. [38]
*Bradyrhizobium liaoningense* LMG 18230^T^	DSM 24092^T^=CECT 4845^T^=USDA 3622^T^	*Glycine max*	China	Xu *et al*. [9]
*Bradyrhizobium yuanmingense* CCBAU 10071^T^	CFNEB 101^T^=CIP 108027^T^=NBRC 100594^T^	*Lespedeza* species	China	Yao *et al*. [59]

The strains are deposited at the Diazotrophic and Plant Growth Promoting Bacteria Culture Collection of Embrapa Soja (WFCC Collection No. 1213, WDCM Collection No. 1054), in Londrina, State of Parana, Brazil; at the Western Australian Soil Microbiology Gene Bank (WSM Culture Collection); at the Belgian Coordinated Collections of Microorganisms (BCCM/LMG); and in other culture collections.

The strains were short-term maintained on modified yeast extract–mannitol agar (YMA) medium [25] at 4 °C in a cold room and periodically cultured, while for long-term preservation the cultures were stored in modified-YM with 30 % glycerol (v/v) at −80 and −150 °C by cryopreservation, and lyophilized as previously described [26].

## Phylogeny

Sequences of the 16S rRNA and of four housekeeping genes (*dnaK*, *glnII*, *gyrB* and *recA*), as well as of the symbiotic gene *nodC* were obtained from a previous study [16], and their accession numbers are shown in Table S1. Amplicons for the housekeeping gene *atpD* were obtained using the pair of primers TSatpDf (5′-TCTGGTCCGYGGCCAGGAAG-3′) and TSatpDr (5′-CGACACTTCCGARCCSGCCTG-3′), with the conditions described by Stepkowski *et al*. [27]. The PureLink kit (Invitrogen) was used following the manufacturer’s instructions for the purification of the PCR products, which were sequenced in an ABI 3500XL (Applied Biosystems) capillary sequencer analyzer, as described by Delamuta *et al*. [28]. All sequences were deposited at the GenBank database (NCBI) and the accession numbers are listed in parentheses in the phylogenetic trees and in Table S1. The complete sequences of housekeeping genes *atpD*, *dnaK*, *glnII*, *gyrB*, *recA* and *rpoB* were also retrieved from the genome of the type strains CNPSo 4010^T^, CNPSo 4016^T^ and CNPSo 4019^T^ in order to build a robust multilocus sequence analysis (MLSA) phylogeny. Multiple sequence alignments were obtained with muscle [29] and the best evolutionary distance model was inferred by the lowest Bayesian information criterion scores [30] for maximum-likelihood (ML) reconstructions in Molecular Evolutionary Genetics Analysis (mega) software version 7 [31]. The evolutionary models are described in the figure captions. The statistical support of the trees was estimated by bootstrap analysis [32] with 1000 re-samplings [33]. *Xanthobacter autotrophicus* Py2 was used as an outgroup for all phylogenies, except for the *nodC* tree. The sequences of housekeeping genes were concatenated with SeaView software version 4.7 [34] for the MLSA. Nucleotide identity was calculated with BioEdit software version 7.0.4.1 [35] for single and concatenated genes and the values are indicated in Table 2.

**Table 2.** Nucleotide identity among new lineages of *Bradyrhizobium* and closely related species, based on the sequences of single and concatenated housekeeping genes (*atpD*, *dnaK*, *glnII*, *gyrB*, *recA* and *rpoB*) and the 16S rRNA

	Nucleotide identity (%)								
**Strains**	**MLSA** (**five genes**)	**MLSA** (**six genes**)	**16S rRNA**	***atpD***	***dnaK***	***glnII***	***gyrB***	***recA***	***rpoB***
	***Bradyrhizobium agreste* CNPSo 4010^T^**								
*B. glycinis* CNPSo 4016^T^	97.2	97.5	100	97.7	98.1	97.4	96.8	95.5	98.6
*B. yuanmingense* CCBAU 10071^T^	96.4	96.9	99.6	94.9	99	95.8	95.3	95.8	98
*B. liaoningense* LMG 18230^T^	95.3	95.3	99.6	94.1	95.9	94.6	94.4	94.7	97.8
*B. diversitatis* CNPSo 4019^T^	95.1	95.3	99.6	94.1	95.9	94.4	94.8	93.8	97.5
*B. frederickii* CNPSo 4026^T^	93.6	94.7	99.5	94.6	95.4	93.8	91.3	93.8	95.6
	***Bradyrhizobium glycinis* CNPSo 4016^T^**								
*B. agreste* CNPSo 4010^T^	97.2	97.5	100	97.7	98.1	97.4	96.8	95.5	98.6
*B. yuanmingense* CCBAU 10071^T^	96.1	97.1	99.6	94.1	98.1	95.8	94.1	96.3	98.3
*B. diversitatis* CNPSo 4019^T^	95.2	95.1	99.6	93.9	96.8	94.8	93.9	94.7	97.2
*B. liaoningense* LMG 18230^T^	94.9	95.1	99.6	94.1	96.8	94.2	93.9	94.4	97
*B. frederickii* CNPSo 4026^T^	93.6	94.5	99.5	95.1	95	94.2	90.7	93.8	95.9
	***Bradyrhizobium diversitatis* CNPSo 4019^T^**								
*B. liaoningense* LMG 18230^T^	97.6	97.8	100	96.9	98.6	94.8	98.1	98.6	99.1
*B. yuanmingense* CCBAU 10071^T^	95.4	95.3	99.5	93.4	95.9	94.4	94.4	95.5	97.8
*B. glycinis* CNPSo 4016^T^	95.2	95.1	99.6	94.1	95.9	94.4	94.8	93.8	97.5
*B. agreste* CNPSo 4010^T^	95.1	95.3	99.6	93.9	96.8	94.8	93.9	94.7	97.2
*B. frederickii* CNPSo 4026^T^	94.8	95.7	99.7	95.4	96.3	93.8	93.9	94.7	97

**Fig. 1.**
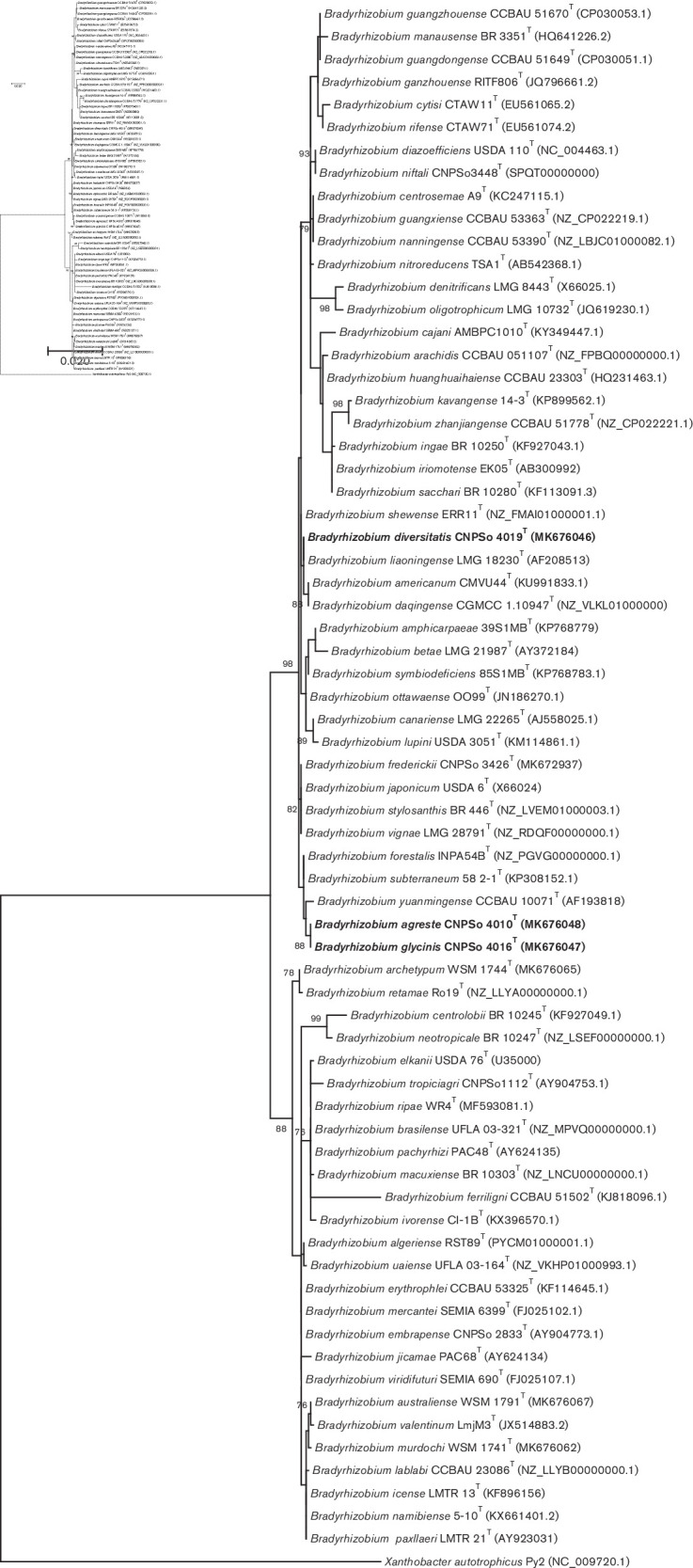
Maximum-likelihood phylogeny based on 16S rRNA alignment (1312 bp), using the T92 (Tamura three-parameter+G+I) model by mega version 7. Accession numbers are indicated in parentheses and in Table S1. The novel species are shown in bold. Bootstrap values >70 % are indicated at the nodes. *Xanthobacter autotrophicus* Py2 was used as an outgroup. Bar indicates two substitutions per 100 nucleotide positions.

The 16S rRNA phylogeny traditionally splits the genus *Bradyrhizobium* into two well-supported superclades, *B. japonicum* and *B. elkanii* [36–38]. We included sequences of all described *Bradyrhizobium* species described at the time of writing and the three strains from our study fit into the *B. japonicum* superclade; strains CNPSo 4010^T^ and CNPSo 4016^T^ showed higher relatedness with *B. yuanmingense* CCBAU 10071^T^ (99.6 % of similarity), whereas the CNPSo 4019^T^ showed 100 % similarity to *B. liaoningense* LMG 18230^T^ and 99.8 % to *B. daqingense* CGMCC 1.10947^T^ (Fig. 1). The nucleotide identity values of the 16S rRNA genes among strains CNPSo 4010^T^, CNPSo 4016^T^ and CNPSo 4019^T^ ranged from 99.6 to 100% (Table 2). Considering the threshold of 98.65 % for species boundary suggested by Kim *et al*. [39], these results corroborate that the 16S rRNA gene provides limited taxonomic information in the genus *Bradyrhizobium* [15, 40–42].

**Fig. 2.**
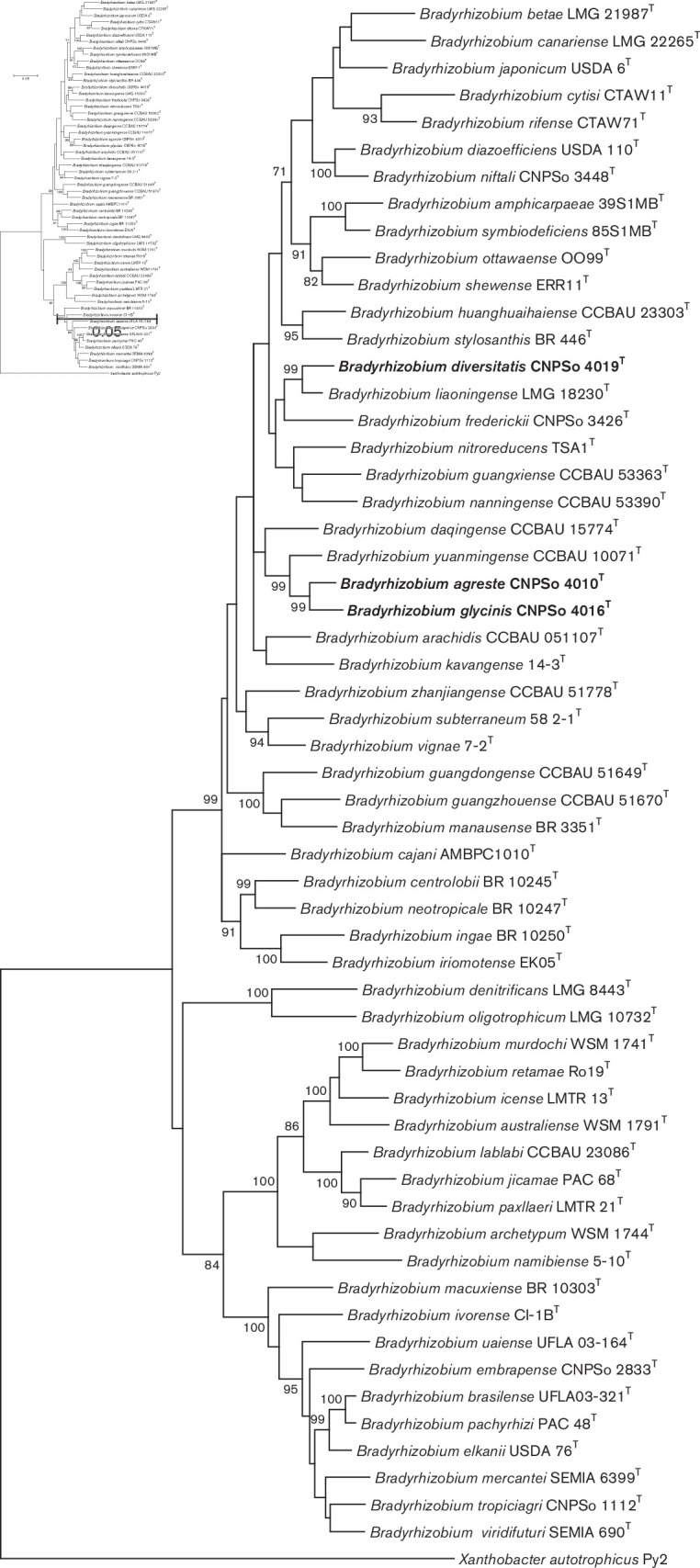
Maximum-likelihood phylogeny based on alignment of *dnaK+glnII+gyrB+recA+rpoB* concatenated genes (2009 bp), using the GTR (general time reversible)+G+I model by mega version 7. Accession numbers are indicated in Table S1. The novel species are shown in bold. Bootstrap values >70 % are indicated at the nodes. *Xanthobacter autotrophicus* Py2 was used as an outgroup. Bar indicates five substitution per 100 nucleotide positions.

In the analysis of housekeeping genes, it is important to verify each single phylogeny to detect congruence with the 16S rRNA phylogeny and possible events of horizontal gene transfer and recombination [36, 43]. Therefore, single phylogenies of the genes *atpD* (398 bp), *dnaK* (221 bp), *glnII* (504 bp), *gyrB* (553 bp), *recA* (360 bp) and *rpoB* (371 bp) were built and are shown in Figs S1–S6 (available in the online version of this article). All phylogenies of single housekeeping genes clustered the strains from this study in clades separated from all described *Bradyrhizobium* species. However, some differences in the topology of the trees were verified on *atpD* (Fig. S1) and *glnII* (Fig. S3) phylogenies. The well-supported *B. japonicum* and *B. elkanii* superclades were not detected in the *atpD* phylogeny, as previously reported by Menna *et al*. [36]. Therefore, in order to improve the phylogenetic information of strains CNPSo 4010^T^, CNPSo 4016^T^ and CNPSo 4019^T^, two alignments of concatenated housekeeping genes were performed for the MLSA; the first including *dnaK+glnII+gyrB+recA+rpoB* genes (2009 bp), and the second with complete sequences of *atpD+dnaK+ glnII+gyrB+recA+rpoB* genes (11 704 bp), and they are shown in Figs 2 and S7, respectively. The resulting concatenated phylograms maintained the main groups of strains observed in the 16S rRNA phylogeny, but improved the species delineation. Also, the similar topology of both MLSA trees demonstrated that this approach represents a reliable buffer against possible events of recombination at a single locus [44].

In the MLSA with five housekeeping genes (Fig. 2), strains CNPSo 4010^T^ and CNPSo 4016^T^ remained in a consistent cluster with 99 % bootstrap support, with *B. yuanmingense* CCBAU 10071^T^ as the closest species with 96.4 and 96.1 % nucleotide identity, respectively (Fig. 2, Table 2). Concerning strain CNPSo 4019^T^, *B. liaoningense* LMG 18230^T^ was the closest species with 99 % bootstrap support and 97.6 % similarity; another close species was *B. frederickii* CNPSo 3426^T^, sharing 94.8 % nucleotide identity. In addition, the nucleotide identity values among strains CNPSo 4010^T^, CNPSo 4016^T^ and CNPSo 4019^T^ ranged from 95.1 to 97.2 %.

Based on an MLSA with five housekeeping genes, Durán *et al*. [45] proposed the threshold of 97 % similarity for *Bradyrhizobium* species delineation. Even though the strains of this study showed nucleotide identity values slightly above this threshold in the MLSA, other species also shared nucleotide identity values higher than 97 %; for example, *B. amphicarpaeae* 39S1MB^T^ and *B. symbiodeficiens* 85S1MB^T^ (97.1 %), *B. elkanii* USDA 76^T^ and *B. pachyrhizi* PAC 48^T^ (97.5 %), *B. elkanii* USDA 76^T^ and *B. brasilense* UFLA03-321^T^ (97.6 %) (data not shown). Recently, also in studies with *Bradyrhizobium*, Klepa *et al*. [15], Helene *et al*. [46] and Fossou *et al*. [47] reported values higher than 97 % when considering five concatenated housekeeping genes. Therefore, as the nucleotide identity is a mathematic parameter and does not take into account specific mutations on gene sequences, we suggest that the threshold of *Bradyrhizobium* species delineation should be revised.

**Fig. 3.**
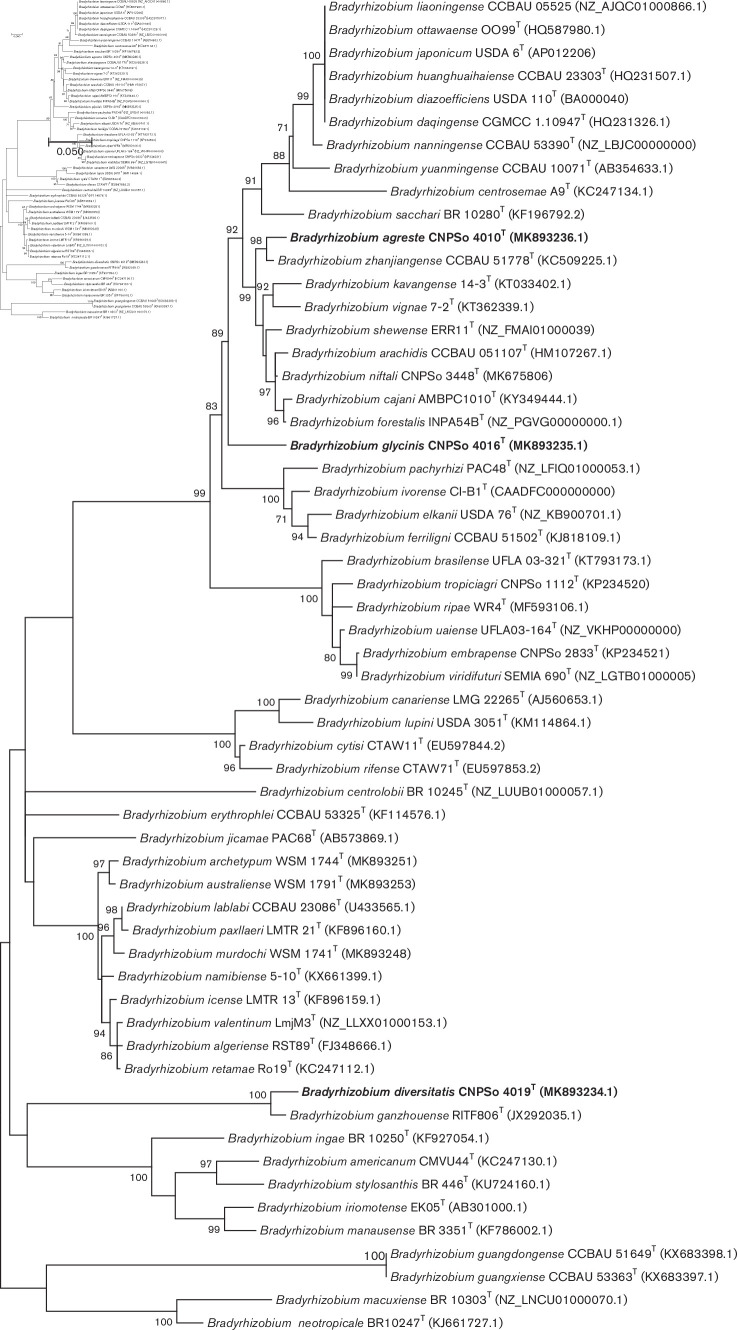
Maximum-likelihood phylogeny based on *nodC* alignment (426 bp), using the T92 (Tamura three-parameter+G+I) model by mega version 7. Accession numbers are indicated in parentheses. The novel species are shown in bold. Bootstrap values >70 % are indicated at the nodes. Bar indicates five substitutions per 100 nucleotide positions.

The symbiotic process between rhizobia and legumes relies on a complex molecular signal exchange that activates several genes responsible for nodule development [48–50]. Phylogeny of nodulation genes does not elucidate the taxonomic status; however, it is important to reveal the evolutionary history of symbiotic relationships, host-range specificity and symbiovar definitions [3, 28, 46, 51, 52]. In this study, the *nodC* gene analysis revealed that CNPSo 4010^T^ from *G. clandestina* clustered in a well-supported clade with *B. zhanjiangense* CCBAU 51778^T^ isolated from nodules of *Arachis hypogaea* in southeast China [53], CNPSo 4019^T^ from *G. max* clustered with 100 % bootstrap support with *B. ganzhouense* RITF806^T^ isolated from nodules of *Acacia melanoxylon* grown in China [54]; whereas CNPSo 4016^T^, isolated from *G. tabacina*, occupied an isolated position (Fig. 3). Note that the phylogenetic position of each of the three strains in the *nodC* tree was different. Comparing the core 16S rRNA and housekeeping genes with the *nodC* gene phylogenies, a different evolutionary pattern was detected in symbiotic phylogeny, suggesting different evolutionary histories, confirming previous reports of other species within the genus *Bradyrhizobium* [16, 28, 51].

## Genome features

Total DNA of strains CNPSo 4010^T^, CNPSo 4016^T^ and CNPSo 4019^T^ was extracted and used to reconstruct the sequence libraries according to the manufacturer’s protocol of Nextera XT kit. The genome sequencing was performed using the MiSeq platform (Illumina) at Embrapa Soja. *De novo* sequence assemblies were carried out by A5-MiSeq pipeline version 20 140 604. The genomes were annotated with RAST version 2.0 [55], using default parameters. The draft genome of CNPSo 4010^T^ (JACCHP000000000) presented about 7 877 331 bp with 75 contigs and an N50 value of 251 874 bp. The genome size of CNPSo 4016^T^ (JACCHQ000000000) was estimated at 7 794 411 bp, containing 83 contigs and an N50 value of 202 190 bp. The genome of CNPSo 4019^T^ (JACEGD000000000) was estimated at 8 450 368 bp, with 133 contigs and an N50 value of 198 871 bp. The sequence coverages of genomes CNPSo 4010^T^, CNPSo 4016^T^ and CNPSo 4019^T^ were estimated at 95-, 91-, and 94-fold, respectively. Coding DNA sequences were identified as 7883 for CNPSo 4010^T^, as 7987 for CNPSo 4016^T^ and 8603 for CNPSo 4019^T^.

Genomic-based measurements of similarity simplify and ensure robust data for microbial taxonomy as a replacement for the traditional DNA–DNA hybridization (DDH) technique [56, 57]. We used average nucleotide identity (ANI) and the digital DNA–DNA hybridization (dDDH) analyses for species delineation considering the suggested cut-off values of 95–96 and 70 %, respectively [56, 58, 59]. The genomes of strains CNPSo 4010^T^, CNPSo 4016^T^ and CNPSo 4019^T^ were compared with the closest related strains according to the phylogenetic analyses: *B. frederickii* CNPSo 3426^T^ (SPQS00000000), *B. liaoningense* LMG 18230^T^ (project ID: 1052895 - JGI) and *B. yuanmingense* CCBAU 10071^T^ (FMAE01000000). ANI values were calculated using the ANI calculator [60] and the dDDH values were estimated with the Genome-to-Genome Distance Calculator (GGDC) version 2.1 [61], using the recommended ‘formula 2’ (identities/HSP length). Strains CNPSo 4010^T^ and CNPSo 4016^T^ shared 94.18 % of ANI and 55.50 % of dDDH to each other and both strains showed values lower than 93.32 % for ANI and 51.80 % for dDDH when compared with *B. yuanmingense* CCBAU 10071^T^ isolated from nodules of *Lespedeza* species plants in China [62] (Table 3). The ANI and dDDH values between CNPSo 4019^T^ and *B. liaoningense* LMG 18230^T^ isolated from nodules of *G. max* grown in China [9] were of 94.97 and 59.80 %, respectively. The values below the threshold for species delineation recorded for the three Australian strains isolated from *Glycine* species confirm that they belong to new species.

**Table 3.** ANI and dDDH values among new lineages of *Bradyrhizobium* and closely related *Bradyrhizobium* species

	* Bradyrhizobium agreste * CNPSo 4010^T^		* Bradyrhizobium glycinis * CNPSo 4016^T^		*Bradyrhizobium diversitatis* CNPSo 4019^T^	
**Strains**	**ANI %**	**dDDH %**	**ANI %**	**dDDH %**	**ANI %**	**dDDH %**
*B. fredereickii* CNPSo 3426^T^ (SPQS00000000)	88.20	35.80	88.13	35.60	88.66	36.90
*B. liaoningense* LMG 18230^T^ (JGI Project Id: 1052895)	89.20	38.40	89.19	38.60	94.97	59.80
*B. yuanmingense* CCBAU 10071^T^ (FMAE01000000)	92.62	48.60	93.32	51.80	89.21	38.40
*B. agreste* CNPSo 4010^T^ (JACCHP000000000)	–	–	94.18	55.50	89.25	38.30
*B. glycinis* CNPSo 4016^T^ (JACCHQ000000000)	94.18	55.50	–	–	89.28	38.50
*B. diversitatis* CNPSo 4019^T^ (JACEGD000000000)	89.26	38.40	89.28	38.40	–	–

The genome G+C contents were calculated using the seed platform [55] and estimated to be 63.8, 63.7 and 63.8 mol% for CNPSo 4010^T^, CNPSo 4016^T^ and CNPSo 4019^T^, respectively.

Genomic diversity at strain level was determined by BOX-PCR, using BOX-A1R primer (5′-CTACGGCAAGGCGACGCTGACG-3′) [63], with the conditions described by Chibeba *et al*. [64]. The BOX-PCR profiles of strains CNPSo 4010^T^, CNPSo 4016^T^ and CNPSo 4019^T^ and the most related species based on the MLSA, *B. liaoningense* LMG 18230^T^ and *B. yuanmingense* CCBAU 10071^T^, were analysed and compared with the Bionumerics software version 7.6 (Applied Mathematics) using the UPGMA (unweighted pair-group method with arithmetic mean) [65] and the Jaccard coefficient [66], with 2 % tolerance. The profiles shared less than 50 % similarity among the strains of this study, confirming high diversity, and also varied in relation to the closest *Bradyrhizobium* species (Fig. S8).

Therefore, this is the first study describing novel species isolated from wild *Glycine* (*G. clandestina* and *G. tabacina*) indigenous to Australia, which are usually neglected compared to the economically important soybean crop. Even though these wild species are not cultivated, they are considered as a secondary genetic source for desirable agronomic traits such as drought tolerance and disease resistance, due the closeness to soybean [17, 20, 22]. Considering the symbiosis with compatible and well-adapted *Bradyrhizobium*, the nodulated wild *Glycine* may represent an interesting option to restore degraded habitats in Australia.

The phylogenetic relationships of the genus *Glycine* have not yet been fully explained. Whereas *G. max* was domesticated in China around 5000 years ago, through the crossing of wild soybean species [67–69], Australia harbours most of the wild species of the genus *Glycine* [17–21]. Some events during the Earth's evolution have been hypothesized to explain the diversification of these legumes. After the Pangea breakup, the Gondwana supercontinent was formed, connecting Australia and Antarctica; these landmasses separated and Australia moved northwards close to Asia. This moment possibly allowed the successfully entry of Asian legumes in Australia and, consequently, their symbionts [14, 70–72].

Taking into account that China is the original centre of soybean, the region might also be a diversification centre for compatible rhizobia [73–75]. The high similarity of CNPSo 4010^T^ and CNPSo 4016^T^ to *B. yuanmingense* CCBAU 10071^T^ and CNPSo 4019^T^ to *B. liaoningense* LMG 18230^T^ may be related to dispersal events. Although *B. yuamingense* CCBAU 10071^T^ was the original symbiont of *Lespezeda* species in China [62], many strains of this species have been isolated from soybean in Asian soils [74–76]. Also, *B. liaoningense* is a widespread soybean symbiont in India and China [9, 77, 78]. Therefore, our findings support the hypothesis of legume and symbionts exchange between Asian and Australian continents.

## Phenotypic characterization

Several morphophysiological evaluations were performed and compared with strains CNPSo 4010^T^, CNPSo 4016^T^ and CNPSo 4019^T^ and closest species *B. liaoningense* LMG 18230^T^ and *B. yuanmingense* CCBAU 10071^T^. Except where indicated, the tests were assessed on modified-YMA medium at 28 °C [25]. Colony morphology was analysed using Congo red and acid and alkaline reaction using bromothymol blue after 7–10 days of growth. In order to evaluate growth under different conditions, the strains were cultured with 1 % NaCl, at 37 °C, on Luria–Bertani (LB) medium, at pH 4.0 and 8.0, adapted from Hungria *et al*. [79]. The urease activity was tested using 2 % urea and phenol red as indicator. Carbohydrate metabolism was detected by the API 50CH kit platform (bioMérieux), according to the manufacturer’s instructions and using modified-YM-minus-mannitol with bromothymol blue. Tolerance of antibiotics was determined by the disc-diffusion technique proposed by Bauer *et al*. [80] using ampicillin (10 µg), bacitracin (10 U), cefuroxime (30 µg), chloramphenicol (30 µg), nalidixic acid (30 µg), neomycin (30 µg), penicillin (10 U), streptomycin (10 µg), tetracycline (30 µg) and erythromycin (15 µg). All tests were conducted in duplicate.

In general, the phenotypic results are in agreement with those commonly found in the genus *Bradyrhizobium*. However, it is worth mentioning some unusual features detected in this study: CNPSo 4010^T^ showed a neutral reaction on modified-YMA with bromothymol blue as indicator and the three strains from this study presented optimal growth on modified-YMA at 37 °C in 3–4 days, indicating a possible mechanism of high temperature tolerance. CNPSo 4019^T^ was able to grow in a large pH range, pH 4.0–8.0, while strain CNPSo 4016^T^ grew well at pH 8.0. Carbohydrate metabolism by API 50CH was heterogeneous among the strains and revealed slightly incongruences with the universal culture medium used to grow rhizobia (YMA), since the CNPSo strains weakly used d-mannitol and also glycerol, another C source commonly used for this culture media. Interestingly, CNPSo 4016^T^ was positive or weak for metabolism of all carbohydrate sources available on the kit. Although our findings revealed different metabolic features, the phenotypic characteristics are generally encoded by the accessory genome, being unstable over time. Also, they may present different results according to the laboratory conditions [81, 82]. The differential phenotypical features among the strains from this study and closest species are reported in Table 4.

**Table 4.** Distinctive phenotypical properties of new lineages of *Bradyrhizobium* and closely related strains Strains: 1, *Bradyrhizobium agreste* CNPSo 4010^T^; 2, *Bradyrhizobium glycinis* CNPSo 4016^T^; 3, *Bradyrhizobium diversitatis* CNPSo 4019^T^; 4, *Bradyrhizobium liaoningense* LMG 18230^T^; 5, *Bradyrhizobium yuanmingense* CCBAU 10071^T^. Data are evaluated as: +, growth; w, weakly positive; −, no growth. na, No data available.

Characteristic	1	2	3	4*	5†
Carbon source utilization:					
Erythritol	−	w	−	−	−
d-Arabinose	w	+	w	+	w
l-Arabinose	w	w	w	+	+
d-Ribose	w	w	w	+	+
d-Xylose	w	+	w	+	+
l-Xylose	w	+	+	+	+
d-Adonitol	−	w	−	−	−
Methyl β-d-xylopyranoside	w	w	w	−	w
d-Galactose	w	w	−	w	w
d-Fructose	w	w	−	−	w
l-Sorbose	w	w	w	−	−
l-Rhamnose	−	w	w	w	w
Dulcitol	−	−	w	−	−
Inositol	−	w	−	−	−
d-Mannitol	w	w	w	−	w
d-Sorbitol	−	w	w	−	−
Methyl α-d-mannopyranoside	−	−	w	−	−
Methyl α-d-glusopyranoside	−	w	w	−	−
*N*-Acetylglucosamine	w	−	w	−	−
Amygdalin	−	+	w	−	−
Arbutin	−	w	w	−	−
Aesculin ferric citrate	w	+	+	+	+
Salicin	−	w	w	−	−
Cellobiose	−	w	w	−	−
Maltose	−	w	w	−	w
Lactose	−	w	w	−	−
Melibiose	−	w	w	−	−
Sucrose	−	w	w	−	−
Trehalose	−	w	w	w	−
Inulin	−	w	w	−	−
Melezitose	−	w	w	−	−
Raffinose	−	w	w	−	−
Glycogen	+	w	+	+	−
Xylitol	−	w	−	−	−
Gentiobiose	−	w	w	−	−
Turanose	−	+	w	−	−
d-Lyxose	+	+	w	+	+
d-Tagatose	−	w	w	−	−
d-Fucose	w	+	w	+	+
d-Arabitol	w	w	w	w	−
l-Arabitol	−	+	−	−	−
Potassium gluconate	−	+	+	−	+
Potassium 2-ketogluconate	+	+	+	−	+
Potassium 5-ketogluconate	w	+	+	−	+
Enzymatic activity:					
Urease	w	w	+	+	na
Growth at/in:					
pH 4	w	w	+	+	−
pH 8	w	+	+	+	+
Alkaline reaction	w	+	+	+	+
37 °C	+ (4 days)	+ (4 days)	+ (3 days)	−	+
Tolerance to antibiotics:					
Ampicillin (10 µg)	+	−	+	−	w
Bacitracin (10 U)	+	+	+	+	na
Erythromycin (15 µg)	−	−	+	+	+
Neomycin (30 µg)	w	w	+	−	−
Streptomycin (10 µg)	−	−	−	−	+
Tetracycline (30 µg)	w	−	+	+	+

*Data obtained from Urquiaga *et al*. [42].

†Data obtained from Delamuta *et al*. [37]; Yao *et al*. [62] and Grönemeyer *et al*. [84].

Nodulation and nitrogen fixation assays were carried out in Leonard jars with soybean commercial cultivar BRASMAX Potência RR and with the promiscuous papilionoid siratro (*Macroptilium atropurpureum*). Each jar was sterilized with sand, vermiculite (2 : 1, v:v) and the N-free nutrient solution described by Broughton and Dilworth [83]. The strains were cultured in modified-YM medium [25] and inoculated on the seeds (1 ml) after planting. The plants grew under controlled glasshouse conditions for 30 days. All strains were able to form effective red colour nodules with siratro but only strain CNPSo 4019^T^ was able to effectively nodulate soybean, its original host (Fig. S9).

Therefore, the phylogenetic, genomic and phenotypic data accomplished in this study support the proposed descriptions of the novel species *Bradyrhizobium agreste* sp. nov., *Bradyrhizobium glycinis* sp. nov. and *Bradyrhizobium diversitatis* sp. nov., isolated from three different *Glycine* species, for which the type strains are CNPSo 4010^T^, CNPSo 4016^T^ and CNPSo 4019^T^, respectively.

## Description of *Bradyrhizobium agreste* sp. nov.

*Bradyrhizobium agreste* (a.gres'te. L. neut. adj. *agreste*, wild, referring to the importance of isolation from wild species, such as the *Glycine clandestina* from this study).

Cells are Gram-stain-negative, aerobic and non-spore-forming. Colonies on modified-YMA medium at pH 6.8–7.0 and Congo red are slightly pink, less than 1 mm in diameter, circular, opaque, with low mucus production and a gummy consistency after 7 days of growth at 28 °C. The strain shows a neutral reaction on modified-YMA with bromothymol blue as indicator and weak urease activity. CNPSo 4010^T^ shows weak growth at pH 4.0 and 8.0. The strain is able to grow at 37 °C for 4 days, but unable to grow on solid LB medium and modified-YMA containing 1 % NaCl. With respect to carbon sources in the API test, the strain is able to use starch, glycogen, d-se, l-fucose and potassium 2-ketogluconate; weakly uses glycerol, d-arabinose, l-arabinose, d-ribose, d-xylose, l-xylose, methyl β-d-xylopyranoside, d-galactose, d-glucose, d-fructose, d-mannose, l-sorbose, d-mannitol, *N*-acetylglucosamine, aesculin ferric citrate, d-fucose, d-arabitol and potassium 5-ketogluconate; does not use erythritol, d-adonitol, l-rhamnose, dulcitol, inositol, d-sorbitol, methyl α-d-mannopyranoside, methyl α-d-glucopyranoside, amygdalin, arbutin, salicin, cellobiose, maltose, lactose, melibiose, sucrose, trehalose, inulin, melezitose, raffinose, xylitol, gentiobiose, turanose, d-tagatose, l-arabitol and potassium gluconate. The strain is tolerant to the antibiotics ampicillin (10 µg), bacitracin (10 U), chloramphenicol (30 µg), nalidixic acid (30 µg) and penicillin G (10 U), moderately sensitive to neomycin (30 µg) and tetracycline (30 µg) and sensitive to cefuroxime (30 µg), erythromycin (15 µg) and streptomycin (10 µg). The strain is able to form effective nitrogen-fixing nodules with *Macroptilium atropurpureum*, but does not nodulate *Glycine max*.

The type strain, CNPSo 4010^T^ (=WSM 4802^T^=LMG 31645^T^), was isolated from a nodule of *Glycine clandestina* in Kununurra, Australia. The DNA G+C content of strain CNPSo 4010^T^ is 63.8 mol%.

## Description of *Bradyrhizobium glycinis* sp. nov.

*Bradyrhizobium glycinis* (gly.ci’nis. N.L. gen. n. *glycinis*, of the genus *Glycine*, a genus that encompasses host plants of several *Bradyrhizobium* species, including this new species, isolated from *G. tabacina*).

Cells are Gram-stain-negative, aerobic and non-spore-forming. Colonies on modified-YMA medium at pH 6.8–7.0 and Congo red are slightly pink, less than 1 mm in diameter, circular, opaque and exhibit low mucus production with a gummy consistency after 7 days of growth at 28 °C. The strain shows an alkaline reaction on modified-YMA with bromothymol blue and weak urease activity. CNPSo 4016^T^ shows weak growth at pH 4.0 but grows well at pH 8.0 and at 37 °C after 4 days. The strain is unable to grow on solid LB medium and modified-YMA containing 1 % NaCl. With respect to carbon sources in the API test, strain CNPSo 4016^T^ is able to use d-arabinose, amygdalin, aesculin ferric citrate, starch, turanose, d-ose, d-fucose, l-fucose, l-arabitol, potassium gluconate, potassium 2-ketogluconate and potassium 5-ketogluconate; weakly uses glycerol, erythritol, l-arabinose, d-ribose, d-xylose, l-xylose, d-adonitol, methyl β-d-xylopyranoside, d-galactose, d-glucose, d-fructose, d-mannose, l-sorbose, l-rhamnose, dulcitol, inositol, d-mannitol, d-sorbitol, methyl α-d-mannopyranoside, methyl α-d-glucopyranoside, *N*-acetylglucosamine, arbutin, salicin, cellobiose, maltose, lactose, melibiose, sucrose, trehalose, inulin, melezitose, raffinose, glycogen, xylitol, gentiobiose, d-tagatose and d-arabitol. The strain is tolerant to bacitracin (10 U), chloramphenicol (30 µg), nalidixic acid (30 µg) and penicillin G (10 U), moderately sensitive to neomycin (30 µg) and sensitive to ampicillin (10 µg), cefuroxime (30 µg), erythromycin (15 µg), streptomycin (10 µg) and tetracycline (30 µg). The strain is able to form effective nitrogen-fixing nodules with *Macroptilium atropurpureum* but does not nodulate *Glycine max*.

The type strain CNPSo 4016^T^ (=WSM 4801^T^=LMG 31649^T^) was isolated from a nodule of *Glycine tabacina* in Kununurra, Australia. The DNA G+C content of strain CNPSo 4016^T^ is 63.7 mol%.

## Description of *Bradyrhizobium diversitatis* sp. nov.

*Bradyrhizobium diversitatis* (di.ver.si.ta’tis. L. fem. n. *diversitas* diversity, N.L. gen. n. *diversitatis*, of diversity, referring to the importance of studies on microbial diversity revealing, as in the case of this study, genetic richness of *Bradyrhizobium* isolated from *Glycine max*).

Cells are Gram-stain-negative, aerobic and non-spore-forming. Colonies on modified-YMA medium at pH 6.8–7.0 and Congo red are slightly pink, less than 1 mm in diameter, circular, opaque and exhibit low mucus production with a gummy consistency after 7 days of growth at 28 °C. The strain shows alkaline reaction on modified-YMA with bromothymol blue, is urease-positive and able to grow at pH 4.0 and 8.0. CNPSo 4019^T^ is able to grow at 37 °C after 3 days. The strain is unable to grow on solid LB medium and modified-YMA containing 1 % NaCl. With respect to carbon sources in the API test, strain CNPSo 4019^T^ is able to use l-xylose, aesculin ferric citrate, starch, glycogen, l-fucose, potassium gluconate, potassium 2-ketogluconate, and potassium 5-ketogluconate; weakly uses glycerol, d-arabinose, l-arabinose, d-ribose, d-xylose, d-adonitol, methyl β-d-xylopyranoside, d-glucose, d-mannose, l-sorbose, l-rhamnose, dulcitol, inositol, d-mannitol, d-sorbitol, methyl α-d-mannopyranoside, methyl α-d-glucopyranoside, *N*-acetylglucosamine, amygdalin, arbutin, salicin, cellobiose, maltose, lactose, melibiose, sucrose, trehalose, inulin, melezitose, raffinose, gentiobiose, turanose, d-lyxose, d-tagatose, d-fucose and d-arabitol; does not use erythritol, d-galactose, d-fructose, xylitol and l-arabitol. The strain is tolerant to the antibiotics ampicillin (10 µg), bacitracin (10 U), chloramphenicol (30 µg), erythromycin (15 µg), nalidixic acid (30 µg), neomycin (30 µg), penicillin G (10 U) and tetracycline (30 µg); and is sensitive to cefuroxime (30 µg) and streptomycin (10 µg). The strain is able to form nitrogen-fixing nodules with *Macroptilium atropurpureum* and *Glycine max*.

The type strain, CNPSo 4019^T^ (=WSM 4799^T^=LMG 31650^T^), was isolated from a nodule of *Glycine max* in Nambung, Australia. The DNA G+C content of strain CNPSo 4019^T^ is 63.8 mol%.

## Supplementary Data

Supplementary material 1
