# Supplementary material for: Bradyrhizobium agreste sp. nov., Bradyrhizobium glycinis sp. nov. and Bradyrhizobium diversitatis sp. nov., isolated from a biodiversity hotspot of the genus Glycine in Western Australia
Source: Int J Syst Evol Microbiol. 2021 Mar 12;71(3):004742. doi: 10.1099/ijsem.0.004742 (PMC8375429; doi:10.1099/ijsem.0.004742)
Supplement: Supplementary material 1 [file ijsem-71-4742-s001.pdf]

International Journal of Systematic and Evolutionary Microbiology

SUPPLEMENTARY MATERIAL

***Bradyrhizobium agreste* sp. nov., *Bradyrhizobium glycinis* sp. nov. and *Bradyrhizobium diversitatis* sp. nov., isolated from a biodiversity hotspot of the genus *Glycine* in Western Australia**

Milena Serenato Klepa, Luisa Caroline Ferraz Helene, Graham O'Hara, Mariangela Hungria*

*Corresponding author: Mariangela Hungria, Embrapa Soja, Cx. Postal 231, 86001-970, Londrina-PR, Brazil. E-mail: mariangela.hungria@embrapa.br; biotecnologia.solo@hotmail.com

Table S1. Accession numbers of 16S rRNA, *atpD*, *dnaK*, *glnII*, *gyrB*, *recA* and *rpoB* used in this study.

Strains	16S	*atpD*	*dnaK*	*glnII*	*gyrB*	*recA*	*rpoB*
B. agreste CNPSo 4010^T	MK676048	MT683853	MK674804	MK860831	MK860852	MK863432	JACCHP000000000
B. glycinis CNPSo 4016^T	MK676047	MT683854	MK674803	MK860830	MK860851	MK863431	JACCHQ000000000
B. diversitatis CNPSo 4019^T	MK676046	MT683855	MK674802	MK860829	MK860850	MK863430	JACEGD000000000
B. algeriense RST89^T	PYCM01000001.1	KF956544.1	FJ264922.1	FJ264924.1	PYCM01000001.1	FJ264927.1	-
B. americanum CMVU44^T	KU991833.1	KC247125.1	-	KX012942.1	-	KC247141.1	-
B. amphicarpaeae 39S1MB^T	KP768779	KP768547.1	CP029426	KP768605.1	KP768721.1	KF615002.1	KP768663.1
B. arachidis CCBAU 051107^T	NZ_FPBQ00000000.1	HM107217.1	JX437668.1	HM107251.1	JX437675.1	HM107233.1	JX437682.1
B. archetypum WSM 1744^T	MK676065	MT070745	MK674820	MK860847	MK860868	MK863448	JAAVLW000000000
B. australiense WSM 1791^T	MK676067	MT070746	MK674822	MK860849	MK860870	MK863450	JAAVLX000000000
B. betae LMG 21987^T	AY372184	FM253129	AY923046.1	AB353733.1	FM253217	AB353734.1	FM253260
B. brasilense UFLA03-321^T	NZ_MPVQ00000000.1	KF452730.1	KF452791.1	NZ_MPVQ00000000	KF452827.1	KT793142.1	KF452879.1
B. cajani AMBPC1010^T	KY349447.1	NZ_WQNE01000000	NZ_WQNE00000000	KY349442.1	NZ_WQNE00000000	KY349440.1	NZ_WQNE01000005.1
B canariense LMG 22265^T	AJ558025.1	AY386739.1	AY923047.1	AY386765.1	FM253220	FM253177	FM253263
B. centrolobii BR 10245^T	KF927049.1	NZ_LUUB01000000	KX527928.1	KX527991.1	KX528004.1	KX527954.1	KF983827.3
B. centrosemae A9^T	KC247115.1	KC247129.1	-	KX012940.1	-	KC247145.1	-
B. cytisi CTAW11^T	EU561065.2	GU001613.1	JQ945184.1	GU001594.1	JN186292.1	GU001575.1	JN186288.1
B. daqingense CGMCC 1.10947^T	NZ_VLKL01000000	HQ231289.1	KF962684.1	HQ231301.1	JX437669.1	HQ231270.1	JX437676.1
B. denitrificans LMG 8443^T	X66025.1	FM253153.1	KF962685.1	HM047121.1	FM253239.1	FM253196.1	FM253282.1
B. diazoefficiens USDA 110^T	NC_004463.1	NC_004463.1	NC_004463.1	NC_004463.1	NC_004463.1	NC_004463.1	NC_004463.1
B. elkanii USDA 76^T	U35000	AY386758.1	AY328392.1	AY599117.1	AM418800	AY591568.1	AM295348
B. embrapense CNPSo 2833^T	AY904773.1	HQ634875	KP234519.2	GQ160500	HQ634891	HQ634899	HQ634910.1
B. erythrophlei CCBAU 53325^T	KF114645.1	-	MG811656.1	KF114693.1	KF114717.1	KF114669.1	MG811654.1

B. ferriligni CCBAU 51502T	KJ818096.1	-	MG811657.1	KJ818099.1	KJ818102.1	KJ818112.1	MG811655.1
B. forestalis INPA54BT	NZ_PGVG00000000.1	KF452722.1	KF452796.1	NZ_PGVG00000000	KF452831.1	KF452867.1	-
B. frederickii CNPSo 3426T	MK672937	SPQS00000000	SPQS00000000	MK682688	MK682721	MK682710	MK682699.1
B. ganzhouense RITF806T	JQ796661.2	JX277182.1	KP420023.1	JX277110.1	KP420022.1	JX277144.1	-
B. guangdongense CCBAU 51649T	CP030051.1	KC508916.1	KC508964.1	KC509023.1	KC509072.1	KC509269.1	KC509318.1
B. guangxiense CCBAU 53363T	NZ_CP022219.1	KC508926.1	KC508974.1	KC509033.1	KC509082.1	KC509279.1	KC509328.1
B. guangzhouense CCBAU 51670T	CP030053.1	KC508902.1	KC508950.1	KC509008.1	KC509057.1	KC509254.1	CP030053.1
B. huanghuaihaiense CCBAU 23303T	HQ231463.1	HQ231682.1	JX437665.1	HQ231639.1	JX437672.1	HQ231595.1	HQ587647.1
B. icense LMTR 13T	KF896156	KF896192	KF896182	KF896175	KF896201	JX943615	NZ_CP016428.1
B. ingae BR 10250T	KF927043.1	KY753593.1	KF927055.1	KF927067.1	KF927079.1	KF927061.1	KF927073.1
B. iriomotense EK05T	AB300992	AB300994.1	JF308944.1	AB300995	AB300997	AB300996	HQ587646.1
B. ivorense CI-1BT	KX396570.1	CAADFC000000000	MK376326	MH756157	MH756161	MK376330	KX388393.1
B. japonicum USDA 6T	X66024	AM168320	AM168362	AF169582	AM418801	AM182158	AM295349
B. jicamae PAC68T	AY624134	FJ428211	JF308945.1	FJ428204	HQ873309.1	HM047133.1	HQ587647
B. kavangense 14-3T	KP899562.1	KY753592.1	KR259949.1	KM378446.1	KX661397.1	KM378399.1	KM378311.1
B. lablabi CCBAU 23086T	NZ_LLYB00000000.1	GU433473.1	KF962687.1	GU433498.1	JX437670.1	GU433522.1	JX437677
B. liaoningense LMG 18230T	AF208513	AY386752.1	AY923041.1	AY386775.1	FM253223	AY591564.1	FM253266
B. lupini USDA 3051T	KM114861.1	KU738808.1	-	KM114862.1	-	KM114866.1	-
B. macuxiense BR 10303T	NZ_LNCU00000000.1	NZ_LNCU01000024.1	KX527932.1	KX527995.1	KX528008.1	KX527958.1	KX527969.1
B. manausense BR 3351T	HQ641226.2	NZ_LJYG01000000	KF786001.1	KF785986.1	KF786000.1	KF785992.1	KF785998.1
B. mercantei SEMIA 6399T	FJ025102.1	NZ_MKFI01000000	KX690617.1	MK860844	KX690623.1	KX690615.1	NZ_MKFI01000000.1
B. murdochi WSM 1741T	MK676062	MT070743	MK674817	MK860844	MK860865	MK863445	AXAU00000000
B. namibiense 5-10T	KX661401.2	KX661387.1	KP402058.1	KM378440.1	KX661393.1	KM378377.1	KM378306.1
B. nanningense CCBAU 53390T	NZ_LBJC01000082.1	KC508921.1	KC508969.1	KC509028.1	KC509077.1	KC509274.1	KC509323.1

B. neotropicale BR 10247^T	NZ_LSEF00000000.1	NZ_LSEF01000000	KJ661693.1	KJ661700.1	KJ661707.1	KJ661714.1	KF983829.1
B. niftali CNPSo 3448^T	SPQT00000000.1	SPQT00000000	SPQT00000000	MK675791	MK675794	MK675797	MK675800.1
B. nitroreducens TSA1^T	AB542368.1	NZ_LFJC00000000	NZ_LFJC01000003.1	NZ_LFJC00000000.1	NZ_LFJC00000000.1	NZ_LFJC00000000.1	NZ_LFJC00000000.1
B. oligotrophicum LMG 10732^T	JQ619230.1	JQ619232.1	KF962688.1	JQ619233.1	KF962697.1	JQ619231.1	KF962713.1
B. ottawaense OO99^T	JN186270.1	HQ455212.1	JF308816.1	HQ587750.1	HQ873179.1	HQ587287.1	HQ587518.1
B. pachyrhizi PAC48^T	AY624135	FJ428208	JF308946.1	FJ428201.1	HQ873310.1	HM047130.1	HQ587648.1
B. paxllaeri LMTR 21^T	AY923031	KF896186	AY923038	KF896169	KF896195	JX943617	KP308154.1
B. retamae Ro19^T	NZ_LLYA00000000.1	FJ428208	KF896184.1	KC247108	KF896204.1	KF962711.1	KF962714.1
B. rifense CTAW71^T	EU561074.2	GU001617.1	JQ945187.1	GU001604.1	KC569466.1	GU001585.1	KC569468.1
B. ripae WR4^T	MF593081.1	-	MF593102.1	MF593086.1	MF593094.1	MF593090.1	MF593098.1
B. sacchari BR 10280^T	KF113091.3	KX065107.1	KX065103.1	KX065099.1	LWIG00000000	KX065095.1	-
B. shewense ERR11^T	NZ_FMAI01000001.1	NZ_FMAI01000000.1	NZ_FMAI00000000	JQ809893.1	NZ_FMAI01000013.1	NZ_FMAI01000022.1	JQ810006.1
B. stylosanthis BR 446^T	NZ_LVEM01000003.1	NZ_LVEM00000000	KU724145	KU724148	KU724151	KU724163.1	KU724166
B. subterraneum 58 2-1^T	KP308152.1	KX661391.1	KP308157.1	KM378484.1	KX661396.1	KM378397	KM378349.1
B. symbiodeficiens 85S1MB^T	KP768783.1	KP768551.1	CP029427	KP768609.1	KP768725.1	KF615036.1	KP768667.1
B. tropiciagri CNPSo 1112^T	AY904753.1	FJ390968	FJ391008.1	FJ391048	HQ634890	FJ391168	HQ634909.1
B. uaiense UFLA 03-164^T	NZ_VKHP01000993.1	KF452739.1	KF452780.1	NZ_VKHP00000000	KT793133.1	KT793144.1	NZ_VKHP00000000
B. valentinum LmjM3^T	JX514883.2	JX518561.2	NZ_LLXX01000028.1	JX518575	NZ_LLXX01000044.1	JX518589.2	-
B. vignae 7-2^T	NZ_RDQF00000000.1	KX683215.1	KR259951.1	KM378443.1	KX683216.1	KM378374.1	KM378308.1
B. viridifuturi SEMIA 690^T	FJ025107.1	NZ_LGTB01000039.1	KR149128	KR149131.1	KR149134.1	KR149140	KU724169.1
B. yuanmingense CCBAU 10071^T	AF193818	AY386760.1	AY923039.1	AY386780.1	FM253226	AM168343	FM253269
B. zhanjiangense CCBAU 51778^T	NZ_CP022221.1	KC508911.1	KC508959.1	KC509017.1	KC509066.1	KC509263.1	KC509312.1

Legends of Supplementary Figures

Figure S1. Maximum likelihood phylogeny based from alignment of the *atpD* (398 bp), using the GTR: General Time Reversible +G+I model by MEGA v. 7. Accession numbers are indicated in parentheses and in Table S1. The novel species are shown in bold. Bootstrap values >70 % are indicated at the nodes. *Xanthobacter autotrophicus* Py2 was used as outgroup. Bar indicates five substitution per 100 nucleotide positions.

Figure S2. Maximum likelihood phylogeny based from alignment of the *dnaK* (221 bp), using the T93: Tamura-Nei +G model by MEGA v. 7. Accession numbers are indicated in parentheses and in Table S1. The novel species is shown are bold. Bootstrap values >70 % are indicated at the nodes. *Xanthobacter autotrophicus* Py2 was used as outgroup. Bar indicates five substitutions per 100 nucleotide positions.

Figure S3. Maximum likelihood phylogeny based from alignment of the *glnII* (504 bp), using the GTR: General Time Reversible +G+I model by MEGA v. 7. Accession numbers are indicated in parentheses and in Table S1. The novel species are shown in bold. Bootstrap values >70 % are indicated at the nodes. *Xanthobacter autotrophicus* Py2 was used as outgroup. Bar indicates two substitutions per 100 nucleotide positions.

Figure S4. Maximum likelihood phylogeny based from alignment of the *gyrB* (553 bp), using the GTR: General Time Reversible +G+I model by MEGA v. 7. Accession numbers are indicated in parentheses and in Table S1. The novel species are shown in bold. Bootstrap values >70 % are indicated at the nodes. *Xanthobacter autotrophicus* Py2 was used as outgroup. Bar indicates five substitutions per 100 nucleotide positions.

Figure S5. Maximum likelihood phylogeny based from alignment of the *recA* (360 bp), using the Tamura 3-Parameter +G model by MEGA v. 7. Accession numbers are indicated in parentheses and in Table S1. The novel species are shown in bold. Bootstrap values >70 % are indicated at the nodes. *Xanthobacter autotrophicus* Py2 was used as outgroup. Bar indicates five substitutions per 100 nucleotide positions.

Figure S6. Maximum likelihood phylogeny based from alignment of the *rpoB* (371 bp), using the GTR: General Time Reversible +G model by MEGA v. 7. Accession numbers are indicated in parentheses and in Table S1. The novel species are shown in bold. Bootstrap values >70 % are indicated at the nodes. *Xanthobacter autotrophicus* Py2 was used as outgroup. Bar indicates two substitution per 100 nucleotide positions.

Figure S7. Maximum likelihood phylogeny based from alignment of the *atpD+dnaK+glnII+gyrB+recA+rpoB* concatenated (11,704 bp), using the GTR: General Time Reversible +G+I model by MEGA v. 7. Accession numbers are indicated in parentheses. The novel species are shown in bold. Bootstrap values >70 % are indicated at the nodes. *Xanthobacter*

autotrophicus Py2 was used as outgroup. Bar indicates five substitution per 100 nucleotide positions.

Figure S8. Dendrogram of similarity based on the BOX-PCR profiles of strains in study and closely related *Bradyrhizobium* species, performed with the program Bionumerics (Applied Mathematics, Kortrijk, Belgium, v.7.6) using the UPGMA algorithm (Unweighted Pair-Group Method with Arithmetic mean) and the Jaccard coefficient, with 2 % tolerance.

Figure S9. Siratro with effective red color nodules formed by (a) *B. agreste* CNPSo 4010^{T}, (b) *B. glycinis* CNPSo 4016^{T} and (c) *B. diversitatis* CNPSo 4019^{T}; (d) soybean, the original host plant of *B. diversitatis* CNPSo 4019^{T}, inoculated by the strain; (e) roots of soybean inoculated with *B. diversitatis* CNPSo 4019^{T}. Plants were grown under axenic conditions at the glasshouse.

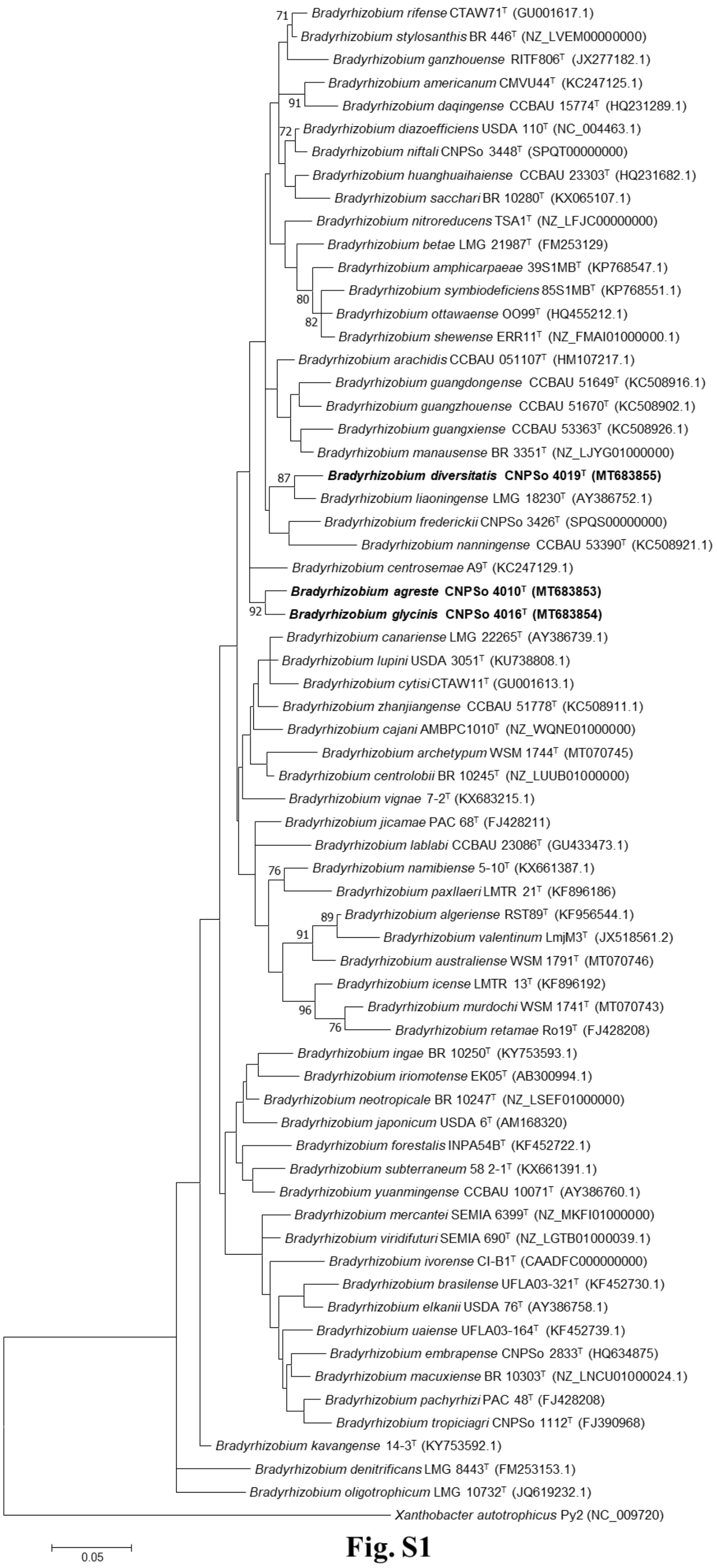

Bradyrhizobium rifense CTAW71T (GU001617.1)
Bradyrhizobium stylosanthis BR 446T (NZ_LVEM00000000)
Bradyrhizobium ganzhouense RITF806T (JX277182.1)
Bradyrhizobium americanum CMVU44T (KC247125.1)
Bradyrhizobium daqingense CCBAU 15774T (HQ231289.1)
Bradyrhizobium diazoefficiens USDA 110T (NC_004463.1)
Bradyrhizobium niftali CNPSo 3448T (SPQT00000000)
Bradyrhizobium huanghuaihaiense CCBAU 23303T (HQ231682.1)
Bradyrhizobium sacchari BR 10280T (KX065107.1)
Bradyrhizobium nitroreducens TSA1T (NZ_LFJC00000000)
Bradyrhizobium betae LMG 21987T (FM253129)
Bradyrhizobium amphicarpaeae 39S1MBT (KP768547.1)
Bradyrhizobium symbiodeficiens 85S1MBT (KP768551.1)
Bradyrhizobium ottawaense OO99T (HQ455212.1)
Bradyrhizobium shewense ERR11T (NZ_FMAI01000000.1)
Bradyrhizobium arachidis CCBAU 051107T (HM107217.1)
Bradyrhizobium guangdongense CCBAU 51649T (KC508916.1)
Bradyrhizobium guangzhouense CCBAU 51670T (KC508902.1)
Bradyrhizobium guangxiense CCBAU 53363T (KC508926.1)
Bradyrhizobium manausense BR 3351T (NZ_LJYG01000000)
Bradyrhizobium diversitatis CNPSo 4019T (MT683855)
Bradyrhizobium liaoningense LMG 18230T (AY386752.1)
Bradyrhizobium frederickii CNPSo 3426T (SPQS00000000)
Bradyrhizobium nanningense CCBAU 53390T (KC508921.1)
Bradyrhizobium centrosemae A9T (KC247129.1)
Bradyrhizobium agreste CNPSo 4010T (MT683853)
Bradyrhizobium glycinis CNPSo 4016T (MT683854)
Bradyrhizobium canariense LMG 22265T (AY386739.1)
Bradyrhizobium lupini USDA 3051T (KU738808.1)
Bradyrhizobium cytisi CTAW11T (GU001613.1)
Bradyrhizobium zhanjiangense CCBAU 51778T (KC508911.1)
Bradyrhizobium cajani AMBPC1010T (NZ_WQNE01000000)
Bradyrhizobium archetypum WSM 1744T (MT070745)
Bradyrhizobium centrolobii BR 10245T (NZ_LUUB01000000)
Bradyrhizobium vignae 7-2T (KX683215.1)
Bradyrhizobium jicamae PAC 68T (FJ428211)
Bradyrhizobium lablabi CCBAU 23086T (GU433473.1)
Bradyrhizobium namibiense 5-10T (KX661387.1)
Bradyrhizobium paxllaeri LMTR 21T (KF896186)
Bradyrhizobium algeriense RST89T (KF956544.1)
Bradyrhizobium valentinum LmjM3T (JX518561.2)
Bradyrhizobium australiense WSM 1791T (MT070746)
Bradyrhizobium icense LMTR 13T (KF896192)
Bradyrhizobium murdochi WSM 1741T (MT070743)
Bradyrhizobium retamae Ro19T (FJ428208)
Bradyrhizobium ingae BR 10250T (KY753593.1)
Bradyrhizobium iriomotense EK05T (AB300994.1)
Bradyrhizobium neotropicale BR 10247T (NZ_LSEF01000000)
Bradyrhizobium japonicum USDA 6T (AM168320)
Bradyrhizobium forestalis INPA54BT (KF452722.1)
Bradyrhizobium subterraneum 58 2-1T (KX661391.1)
Bradyrhizobium yuanmingense CCBAU 10071T (AY386760.1)
Bradyrhizobium mercantei SEMIA 6399T (NZ_MKFI01000000)
Bradyrhizobium viridifuturi SEMIA 690T (NZ_LGTB01000039.1)
Bradyrhizobium ivorense CI-B1T (CAADFC000000000)
Bradyrhizobium brasilense UFLA03-321T (KF452730.1)
Bradyrhizobium elkanii USDA 76T (AY386758.1)
Bradyrhizobium uaiense UFLA03-164T (KF452739.1)
Bradyrhizobium embrapense CNPSo 2833T (HQ634875)
Bradyrhizobium macuxiense BR 10303T (NZ_LNCU01000024.1)
Bradyrhizobium pachyrhizi PAC 48T (FJ428208)
Bradyrhizobium tropiciagri CNPSo 1112T (FJ390968)
Bradyrhizobium kavangense 14-3T (KY753592.1)
Bradyrhizobium denitrificans LMG 8443T (FM253153.1)
Bradyrhizobium oligotrophicum LMG 10732T (JQ619232.1)
Xanthobacter autotrophicus Py2 (NC_009720)
71
91
72
80
82
87
92
76
89
91
96
76
0.05

Fig. S1

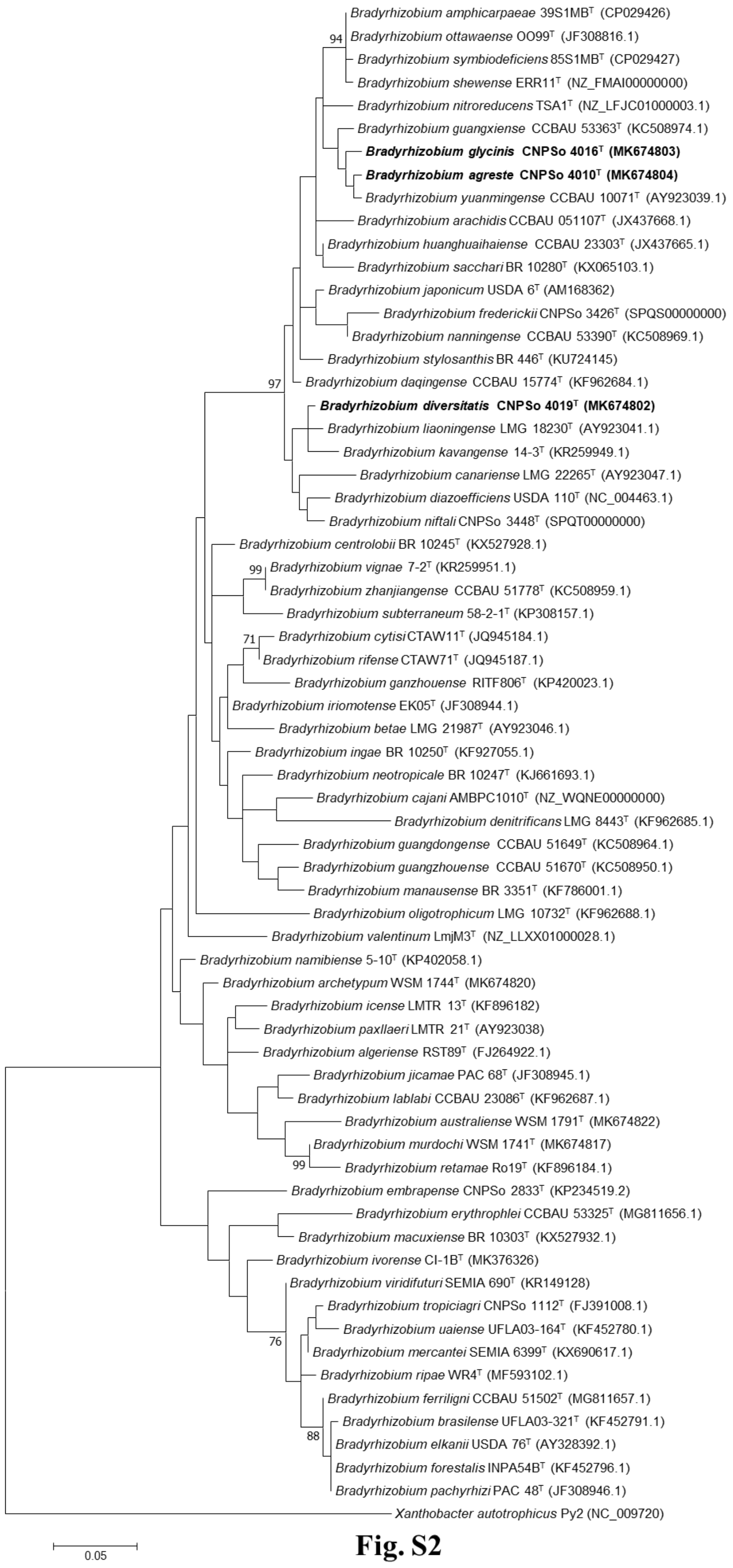
Bradyrhizobium amphicarpaeae 39S1MB^{T} (CP029426)
Bradyrhizobium ottawaense OO99^{T} (JF308816.1)
Bradyrhizobium symbiodeficiens 85S1MB^{T} (CP029427)
Bradyrhizobium shewense ERR11^{T} (NZ_FMAI00000000)
Bradyrhizobium nitroreducens TSA1^{T} (NZ_LFJC01000003.1)
Bradyrhizobium guangxiense CCBAU 53363^{T} (KC508974.1)
Bradyrhizobium glycinis CNPSo 4016^{T} (MK674803)
Bradyrhizobium agreste CNPSo 4010^{T} (MK674804)
Bradyrhizobium yuanmingense CCBAU 10071^{T} (AY923039.1)
Bradyrhizobium arachidis CCBAU 051107^{T} (JX437668.1)
Bradyrhizobium huanghuaihaiense CCBAU 23303^{T} (JX437665.1)
Bradyrhizobium sacchari BR 10280^{T} (KX065103.1)
Bradyrhizobium japonicum USDA 6^{T} (AM168362)
Bradyrhizobium frederickii CNPSo 3426^{T} (SPQS00000000)
Bradyrhizobium nanningense CCBAU 53390^{T} (KC508969.1)
Bradyrhizobium stylosanthis BR 446^{T} (KU724145)
Bradyrhizobium daqingense CCBAU 15774^{T} (KF962684.1)
Bradyrhizobium diversitatis CNPSo 4019^{T} (MK674802)
Bradyrhizobium liaoningense LMG 18230^{T} (AY923041.1)
Bradyrhizobium kavangense 14-3^{T} (KR259949.1)
Bradyrhizobium canariense LMG 22265^{T} (AY923047.1)
Bradyrhizobium diazoefficiens USDA 110^{T} (NC_004463.1)
Bradyrhizobium niftali CNPSo 3448^{T} (SPQT00000000)
Bradyrhizobium centrolobii BR 10245^{T} (KX527928.1)
Bradyrhizobium vignae 7-2^{T} (KR259951.1)
Bradyrhizobium zhanjiangense CCBAU 51778^{T} (KC508959.1)
Bradyrhizobium subterraneum 58-2-1^{T} (KP308157.1)
Bradyrhizobium cytisi CTAW11^{T} (JQ945184.1)
Bradyrhizobium rifense CTAW71^{T} (JQ945187.1)
Bradyrhizobium ganzhouense RITF806^{T} (KP420023.1)
Bradyrhizobium iriomotense EK05^{T} (JF308944.1)
Bradyrhizobium betae LMG 21987^{T} (AY923046.1)
Bradyrhizobium ingae BR 10250^{T} (KF927055.1)
Bradyrhizobium neotropicale BR 10247^{T} (KJ661693.1)
Bradyrhizobium cajani AMBPC1010^{T} (NZ_WQNE00000000)
Bradyrhizobium denitrificans LMG 8443^{T} (KF962685.1)
Bradyrhizobium guangdongense CCBAU 51649^{T} (KC508964.1)
Bradyrhizobium guangzhouense CCBAU 51670^{T} (KC508950.1)
Bradyrhizobium manausense BR 3351^{T} (KF786001.1)
Bradyrhizobium oligotrophicum LMG 10732^{T} (KF962688.1)
Bradyrhizobium valentinum LmjM3^{T} (NZ_LLXX01000028.1)
Bradyrhizobium namibiense 5-10^{T} (KP402058.1)
Bradyrhizobium archetypum WSM 1744^{T} (MK674820)
Bradyrhizobium icense LMTR 13^{T} (KF896182)
Bradyrhizobium paxllaeri LMTR 21^{T} (AY923038)
Bradyrhizobium algeriense RST89^{T} (FJ264922.1)
Bradyrhizobium jicamae PAC 68^{T} (JF308945.1)
Bradyrhizobium lablabi CCBAU 23086^{T} (KF962687.1)
Bradyrhizobium australiense WSM 1791^{T} (MK674822)
Bradyrhizobium murdochi WSM 1741^{T} (MK674817)
Bradyrhizobium retamae Ro19^{T} (KF896184.1)
Bradyrhizobium embrapense CNPSo 2833^{T} (KP234519.2)
Bradyrhizobium erythrophlei CCBAU 53325^{T} (MG811656.1)
Bradyrhizobium macuxiense BR 10303^{T} (KX527932.1)
Bradyrhizobium ivorense CI-1B^{T} (MK376326)
Bradyrhizobium viridifuturi SEMIA 690^{T} (KR149128)
Bradyrhizobium tropiciagri CNPSo 1112^{T} (FJ391008.1)
Bradyrhizobium uaiense UFLA03-164^{T} (KF452780.1)
Bradyrhizobium mercantei SEMIA 6399^{T} (KX690617.1)
Bradyrhizobium ripae WR4^{T} (MF593102.1)
Bradyrhizobium ferriligni CCBAU 51502^{T} (MG811657.1)
Bradyrhizobium brasilense UFLA03-321^{T} (KF452791.1)
Bradyrhizobium elkanii USDA 76^{T} (AY328392.1)
Bradyrhizobium forestalis INPA54B^{T} (KF452796.1)
Bradyrhizobium pachyrhizi PAC 48^{T} (JF308946.1)
Xanthobacter autotrophicus Py2 (NC_009720)
94
97
99
71
99
76
88
0.05

Fig. S2

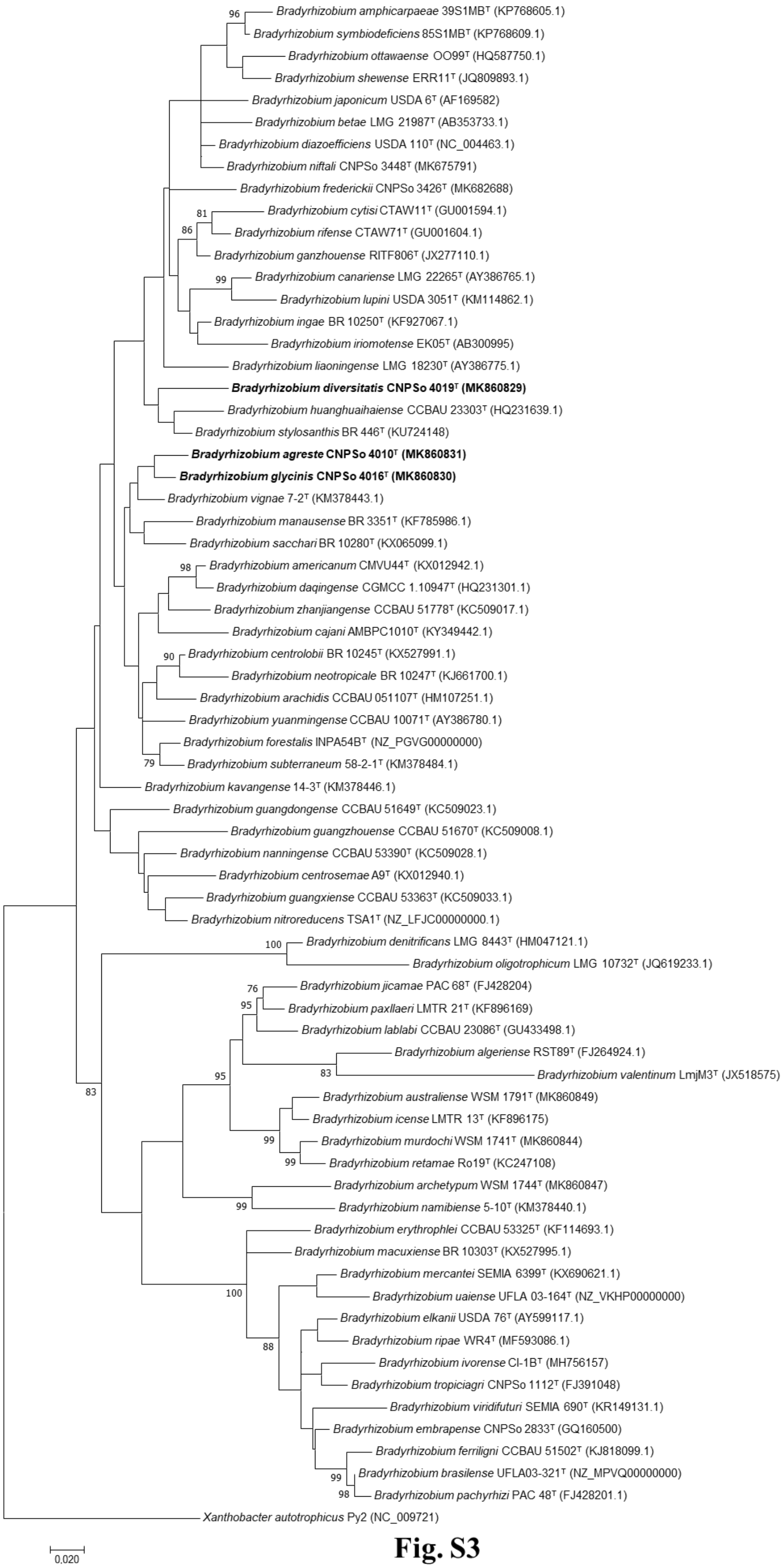
Bradyrhizobium amphicarpaeae 39S1MBT (KP768605.1)
Bradyrhizobium symbiodeficiens 85S1MBT (KP768609.1)
Bradyrhizobium ottawaense OO99T (HQ587750.1)
Bradyrhizobium shewense ERR11T (JQ809893.1)
Bradyrhizobium japonicum USDA 6T (AF169582)
Bradyrhizobium betae LMG 21987T (AB353733.1)
Bradyrhizobium diazoefficiens USDA 110T (NC_004463.1)
Bradyrhizobium niftali CNPSo 3448T (MK675791)
Bradyrhizobium frederickii CNPSo 3426T (MK682688)
Bradyrhizobium cytisi CTAW11T (GU001594.1)
Bradyrhizobium rifense CTAW71T (GU001604.1)
Bradyrhizobium ganzhouense RITF806T (JX277110.1)
Bradyrhizobium canariense LMG 22265T (AY386765.1)
Bradyrhizobium lupini USDA 3051T (KM114862.1)
Bradyrhizobium ingae BR 10250T (KF927067.1)
Bradyrhizobium iriomotense EK05T (AB300995)
Bradyrhizobium liaoningense LMG 18230T (AY386775.1)
Bradyrhizobium diversitatis CNPSo 4019T (MK860829)
Bradyrhizobium huanghuaihaiense CCBAU 23303T (HQ231639.1)
Bradyrhizobium stylosanthis BR 446T (KU724148)
Bradyrhizobium agreste CNPSo 4010T (MK860831)
Bradyrhizobium glycinis CNPSo 4016T (MK860830)
Bradyrhizobium vignae 7-2T (KM378443.1)
Bradyrhizobium manausense BR 3351T (KF785986.1)
Bradyrhizobium sacchari BR 10280T (KX065099.1)
Bradyrhizobium americanum CMVU44T (KX012942.1)
Bradyrhizobium daqingense CGMCC 1.10947T (HQ231301.1)
Bradyrhizobium zhanjiangense CCBAU 51778T (KC509017.1)
Bradyrhizobium cajani AMBPC1010T (KY349442.1)
Bradyrhizobium centrolobii BR 10245T (KX527991.1)
Bradyrhizobium neotropicale BR 10247T (KJ661700.1)
Bradyrhizobium arachidis CCBAU 051107T (HM107251.1)
Bradyrhizobium yuanmingense CCBAU 10071T (AY386780.1)
Bradyrhizobium forestalis INPA54BT (NZ_PGVG00000000)
Bradyrhizobium subterraneum 58-2-1T (KM378484.1)
Bradyrhizobium kavangense 14-3T (KM378446.1)
Bradyrhizobium guangdongense CCBAU 51649T (KC509023.1)
Bradyrhizobium guangzhouense CCBAU 51670T (KC509008.1)
Bradyrhizobium nanningense CCBAU 53390T (KC509028.1)
Bradyrhizobium centrosemae A9T (KX012940.1)
Bradyrhizobium guangxiense CCBAU 53363T (KC509033.1)
Bradyrhizobium nitroreducens TSA1T (NZ_LFJC00000000.1)
Bradyrhizobium denitrificans LMG 8443T (HM047121.1)
Bradyrhizobium oligotrophicum LMG 10732T (JQ619233.1)
Bradyrhizobium jicamae PAC 68T (FJ428204)
Bradyrhizobium paxllaeri LMTR 21T (KF896169)
Bradyrhizobium lablabi CCBAU 23086T (GU433498.1)
Bradyrhizobium algeriense RST89T (FJ264924.1)
Bradyrhizobium valentinum LmjM3T (JX518575)
Bradyrhizobium australiense WSM 1791T (MK860849)
Bradyrhizobium icense LMTR 13T (KF896175)
Bradyrhizobium murdochi WSM 1741T (MK860844)
Bradyrhizobium retamae Ro19T (KC247108)
Bradyrhizobium archetypum WSM 1744T (MK860847)
Bradyrhizobium namibiense 5-10T (KM378440.1)
Bradyrhizobium erythrophlei CCBAU 53325T (KF114693.1)
Bradyrhizobium macuxiense BR 10303T (KX527995.1)
Bradyrhizobium mercantei SEMIA 6399T (KX690621.1)
Bradyrhizobium uaiense UFLA 03-164T (NZ_VKHP00000000)
Bradyrhizobium elkanii USDA 76T (AY599117.1)
Bradyrhizobium ripae WR4T (MF593086.1)
Bradyrhizobium ivorense CI-1BT (MH756157)
Bradyrhizobium tropiciagri CNPSo 1112T (FJ391048)
Bradyrhizobium viridifuturi SEMIA 690T (KR149131.1)
Bradyrhizobium embrapense CNPSo 2833T (GQ160500)
Bradyrhizobium ferriligni CCBAU 51502T (KJ818099.1)
Bradyrhizobium brasilense UFLA03-321T (NZ_MPVQ00000000)
Bradyrhizobium pachyrhizi PAC 48T (FJ428201.1)
Xanthobacter autotrophicus Py2 (NC_009721)
96
81
86
99
98
90
79
100
76
95
83
95
99
99
99
100
88
99
98
83
0,020

Fig. S3

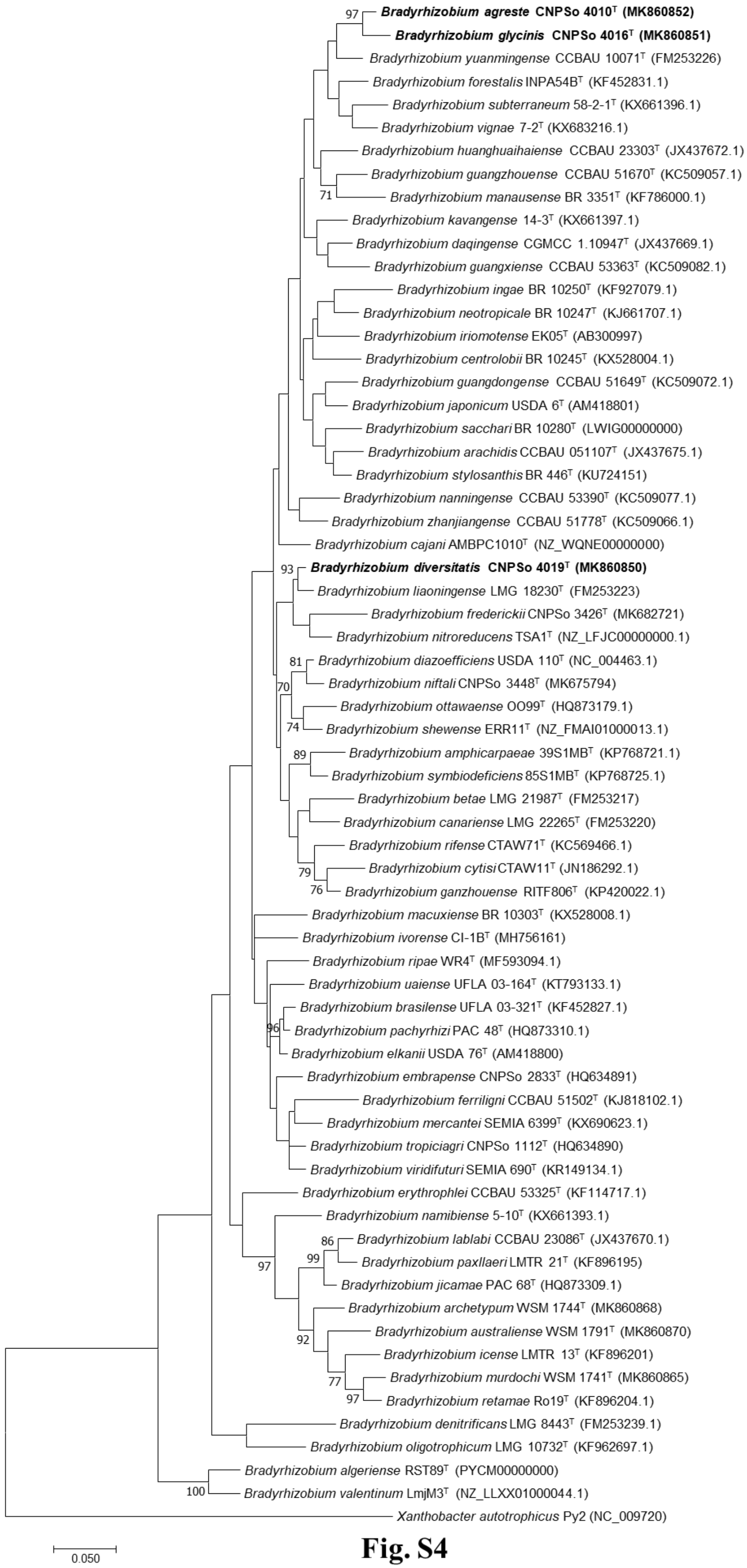
Bradyrhizobium agreste CNPSo 4010^{T} (MK860852)
Bradyrhizobium glycinis CNPSo 4016^{T} (MK860851)
Bradyrhizobium yuanmingense CCBAU 10071^{T} (FM253226)
Bradyrhizobium forestalis INPA54B^{T} (KF452831.1)
Bradyrhizobium subterraneum 58-2-1^{T} (KX661396.1)
Bradyrhizobium vignae 7-2^{T} (KX683216.1)
Bradyrhizobium huanghuaihaiense CCBAU 23303^{T} (JX437672.1)
Bradyrhizobium guangzhouense CCBAU 51670^{T} (KC509057.1)
Bradyrhizobium manausense BR 3351^{T} (KF786000.1)
Bradyrhizobium kavangense 14-3^{T} (KX661397.1)
Bradyrhizobium daqingense CGMCC 1.10947^{T} (JX437669.1)
Bradyrhizobium guangxiense CCBAU 53363^{T} (KC509082.1)
Bradyrhizobium ingae BR 10250^{T} (KF927079.1)
Bradyrhizobium neotropicale BR 10247^{T} (KJ661707.1)
Bradyrhizobium iriomotense EK05^{T} (AB300997)
Bradyrhizobium centrolobii BR 10245^{T} (KX528004.1)
Bradyrhizobium guangdongense CCBAU 51649^{T} (KC509072.1)
Bradyrhizobium japonicum USDA 6^{T} (AM418801)
Bradyrhizobium sacchari BR 10280^{T} (LWIG00000000)
Bradyrhizobium arachidis CCBAU 051107^{T} (JX437675.1)
Bradyrhizobium stylosanthis BR 446^{T} (KU724151)
Bradyrhizobium nanningense CCBAU 53390^{T} (KC509077.1)
Bradyrhizobium zhanjiangense CCBAU 51778^{T} (KC509066.1)
Bradyrhizobium cajani AMBPC1010^{T} (NZ_WQNE00000000)
Bradyrhizobium diversitatis CNPSo 4019^{T} (MK860850)
Bradyrhizobium liaoningense LMG 18230^{T} (FM253223)
Bradyrhizobium frederickii CNPSo 3426^{T} (MK682721)
Bradyrhizobium nitroreducens TSA1^{T} (NZ_LFJC00000000.1)
Bradyrhizobium diazoefficiens USDA 110^{T} (NC_004463.1)
Bradyrhizobium niftali CNPSo 3448^{T} (MK675794)
Bradyrhizobium ottawaense OO99^{T} (HQ873179.1)
Bradyrhizobium shewense ERR11^{T} (NZ_FMAI01000013.1)
Bradyrhizobium amphicarpaeae 39S1MB^{T} (KP768721.1)
Bradyrhizobium symbiodeficiens 85S1MB^{T} (KP768725.1)
Bradyrhizobium betae LMG 21987^{T} (FM253217)
Bradyrhizobium canariense LMG 22265^{T} (FM253220)
Bradyrhizobium rifense CTAW71^{T} (KC569466.1)
Bradyrhizobium cytisi CTAW11^{T} (JN186292.1)
Bradyrhizobium ganzhouense RITF806^{T} (KP420022.1)
Bradyrhizobium macuxiense BR 10303^{T} (KX528008.1)
Bradyrhizobium ivorense CI-1B^{T} (MH756161)
Bradyrhizobium ripae WR4^{T} (MF593094.1)
Bradyrhizobium uaiense UFLA 03-164^{T} (KT793133.1)
Bradyrhizobium brasilense UFLA 03-321^{T} (KF452827.1)
Bradyrhizobium pachyrhizi PAC 48^{T} (HQ873310.1)
Bradyrhizobium elkanii USDA 76^{T} (AM418800)
Bradyrhizobium embrapense CNPSo 2833^{T} (HQ634891)
Bradyrhizobium ferriligni CCBAU 51502^{T} (KJ818102.1)
Bradyrhizobium mercantei SEMIA 6399^{T} (KX690623.1)
Bradyrhizobium tropiciagri CNPSo 1112^{T} (HQ634890)
Bradyrhizobium viridifuturi SEMIA 690^{T} (KR149134.1)
Bradyrhizobium erythrophlei CCBAU 53325^{T} (KF114717.1)
Bradyrhizobium namibiense 5-10^{T} (KX661393.1)
Bradyrhizobium lablabi CCBAU 23086^{T} (JX437670.1)
Bradyrhizobium paxllaeri LMTR 21^{T} (KF896195)
Bradyrhizobium jicamae PAC 68^{T} (HQ873309.1)
Bradyrhizobium archetypum WSM 1744^{T} (MK860868)
Bradyrhizobium australiense WSM 1791^{T} (MK860870)
Bradyrhizobium icense LMTR 13^{T} (KF896201)
Bradyrhizobium murdochi WSM 1741^{T} (MK860865)
Bradyrhizobium retamae Ro19^{T} (KF896204.1)
Bradyrhizobium denitrificans LMG 8443^{T} (FM253239.1)
Bradyrhizobium oligotrophicum LMG 10732^{T} (KF962697.1)
Bradyrhizobium algeriense RST89^{T} (PYCM00000000)
Bradyrhizobium valentinum LmjM3^{T} (NZ_LLXX01000044.1)
Xanthobacter autotrophicus Py2 (NC_009720)
97
71
93
81
70
74
89
79
76
96
86
99
97
92
77
97
100
0.050

Fig. S4

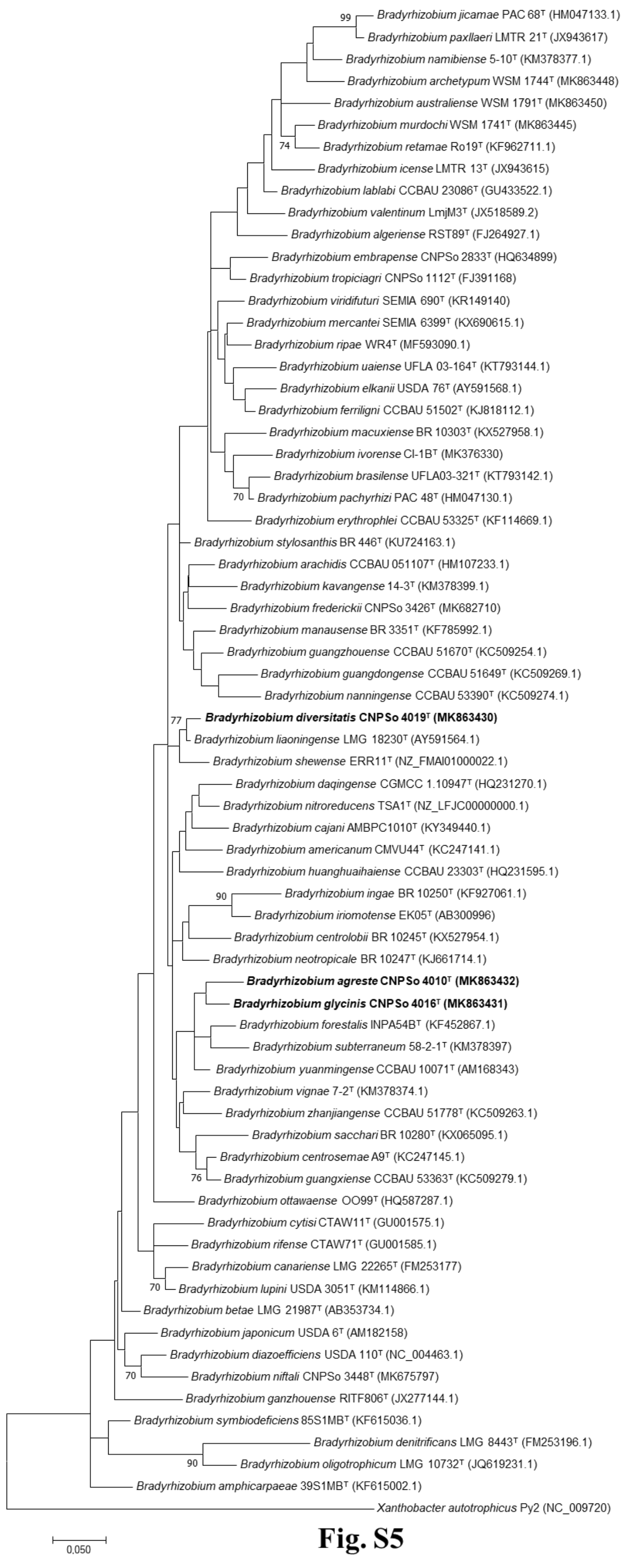
Bradyrhizobium jicamae PAC 68T (HM047133.1)
Bradyrhizobium paxllaeri LMTR 21T (JX943617)
Bradyrhizobium namibiense 5-10T (KM378377.1)
Bradyrhizobium archetypum WSM 1744T (MK863448)
Bradyrhizobium australiense WSM 1791T (MK863450)
Bradyrhizobium murdochi WSM 1741T (MK863445)
Bradyrhizobium retamae Ro19T (KF962711.1)
Bradyrhizobium icense LMTR 13T (JX943615)
Bradyrhizobium lablabi CCBAU 23086T (GU433522.1)
Bradyrhizobium valentinum LmjM3T (JX518589.2)
Bradyrhizobium algeriense RST89T (FJ264927.1)
Bradyrhizobium embrapense CNPSo 2833T (HQ634899)
Bradyrhizobium tropiciagri CNPSo 1112T (FJ391168)
Bradyrhizobium viridifuturi SEMIA 690T (KR149140)
Bradyrhizobium mercantei SEMIA 6399T (KX690615.1)
Bradyrhizobium ripae WR4T (MF593090.1)
Bradyrhizobium uaiense UFLA 03-164T (KT793144.1)
Bradyrhizobium elkanii USDA 76T (AY591568.1)
Bradyrhizobium ferriligni CCBAU 51502T (KJ818112.1)
Bradyrhizobium macuxiense BR 10303T (KX527958.1)
Bradyrhizobium ivorense CI-1BT (MK376330)
Bradyrhizobium brasilense UFLA03-321T (KT793142.1)
Bradyrhizobium pachyrhizi PAC 48T (HM047130.1)
Bradyrhizobium erythrophlei CCBAU 53325T (KF114669.1)
Bradyrhizobium stylosanthis BR 446T (KU724163.1)
Bradyrhizobium arachidis CCBAU 051107T (HM107233.1)
Bradyrhizobium kavangense 14-3T (KM378399.1)
Bradyrhizobium frederickii CNPSo 3426T (MK682710)
Bradyrhizobium manausense BR 3351T (KF785992.1)
Bradyrhizobium guangzhouense CCBAU 51670T (KC509254.1)
Bradyrhizobium guangdongense CCBAU 51649T (KC509269.1)
Bradyrhizobium nanningense CCBAU 53390T (KC509274.1)
Bradyrhizobium diversitatis CNPSo 4019T (MK863430)
Bradyrhizobium liaoningense LMG 18230T (AY591564.1)
Bradyrhizobium shewense ERR11T (NZ_FMAI01000022.1)
Bradyrhizobium daqingense CGMCC 1.10947T (HQ231270.1)
Bradyrhizobium nitroreducens TSA1T (NZ_LFJC00000000.1)
Bradyrhizobium cajani AMBPC1010T (KY349440.1)
Bradyrhizobium americanum CMVU44T (KC247141.1)
Bradyrhizobium huanghuaihaiense CCBAU 23303T (HQ231595.1)
Bradyrhizobium ingae BR 10250T (KF927061.1)
Bradyrhizobium iriomotense EK05T (AB300996)
Bradyrhizobium centrolobii BR 10245T (KX527954.1)
Bradyrhizobium neotropicale BR 10247T (KJ661714.1)
Bradyrhizobium agreste CNPSo 4010T (MK863432)
Bradyrhizobium glycinis CNPSo 4016T (MK863431)
Bradyrhizobium forestalis INPA54BT (KF452867.1)
Bradyrhizobium subterraneum 58-2-1T (KM378397)
Bradyrhizobium yuanmingense CCBAU 10071T (AM168343)
Bradyrhizobium vignae 7-2T (KM378374.1)
Bradyrhizobium zhanjiangense CCBAU 51778T (KC509263.1)
Bradyrhizobium sacchari BR 10280T (KX065095.1)
Bradyrhizobium centrosemae A9T (KC247145.1)
Bradyrhizobium guangxiense CCBAU 53363T (KC509279.1)
Bradyrhizobium ottawaense OO99T (HQ587287.1)
Bradyrhizobium cytisi CTAW11T (GU001575.1)
Bradyrhizobium rifense CTAW71T (GU001585.1)
Bradyrhizobium canariense LMG 22265T (FM253177)
Bradyrhizobium lupini USDA 3051T (KM114866.1)
Bradyrhizobium betae LMG 21987T (AB353734.1)
Bradyrhizobium japonicum USDA 6T (AM182158)
Bradyrhizobium diazoefficiens USDA 110T (NC_004463.1)
Bradyrhizobium niftali CNPSo 3448T (MK675797)
Bradyrhizobium ganzhouense RITF806T (JX277144.1)
Bradyrhizobium symbiodeficiens 85S1MBT (KF615036.1)
Bradyrhizobium denitrificans LMG 8443T (FM253196.1)
Bradyrhizobium oligotrophicum LMG 10732T (JQ619231.1)
Bradyrhizobium amphicarpaeae 39S1MBT (KF615002.1)
Xanthobacter autotrophicus Py2 (NC_009720)
99
74
70
77
90
76
70
70
90
0,050

Fig. S5

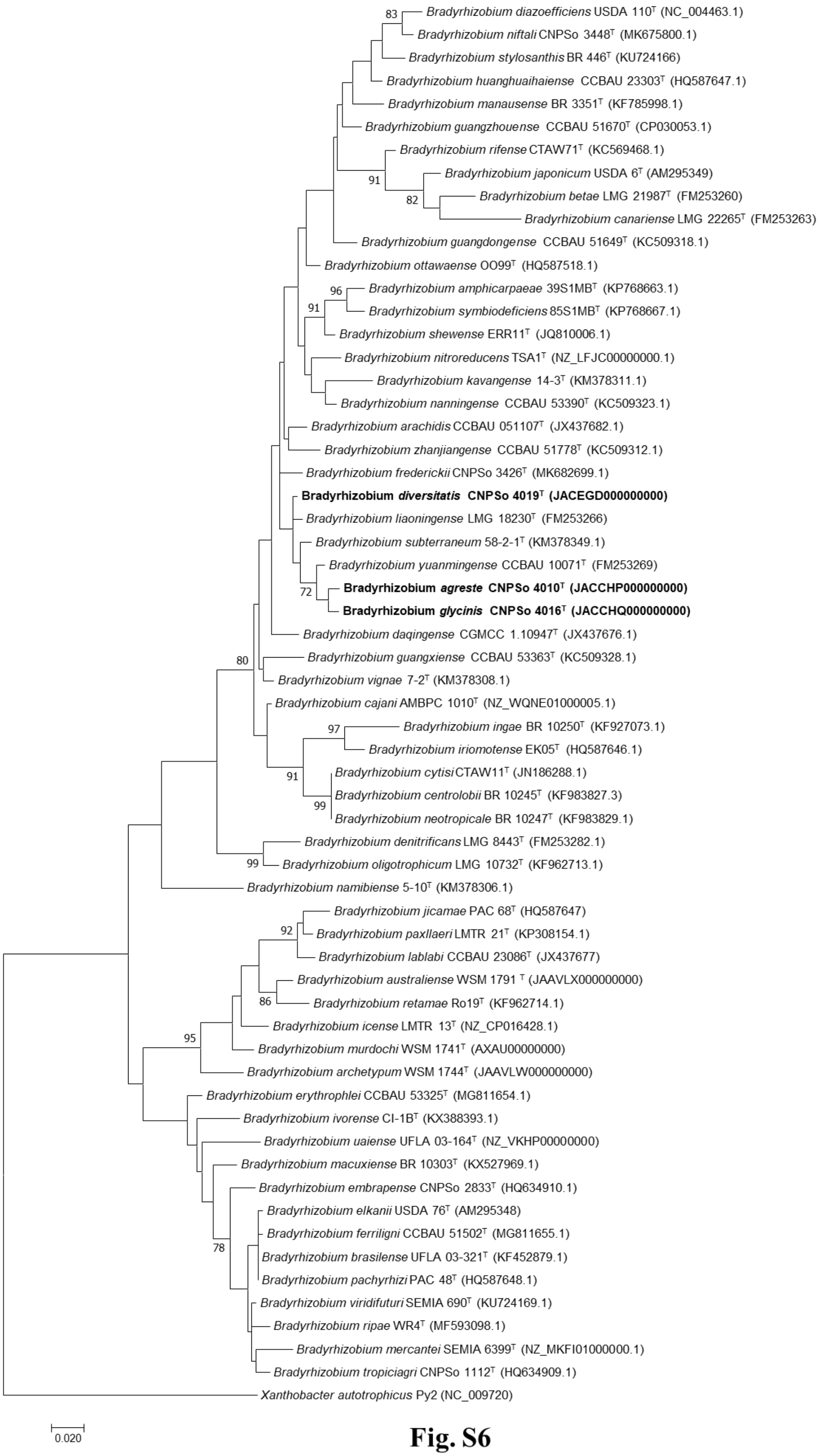

Bradyrhizobium diazoefficiens USDA 110T (NC_004463.1)
Bradyrhizobium niftali CNPSo 3448T (MK675800.1)
Bradyrhizobium stylosanthis BR 446T (KU724166)
Bradyrhizobium huanghuaihaiense CCBAU 23303T (HQ587647.1)
Bradyrhizobium manausense BR 3351T (KF785998.1)
Bradyrhizobium guangzhouense CCBAU 51670T (CP030053.1)
Bradyrhizobium rifense CTAW71T (KC569468.1)
Bradyrhizobium japonicum USDA 6T (AM295349)
Bradyrhizobium betae LMG 21987T (FM253260)
Bradyrhizobium canariense LMG 22265T (FM253263)
Bradyrhizobium guangdongense CCBAU 51649T (KC509318.1)
Bradyrhizobium ottawaense OO99T (HQ587518.1)
Bradyrhizobium amphicarpaeae 39S1MBT (KP768663.1)
Bradyrhizobium symbiodeficiens 85S1MBT (KP768667.1)
Bradyrhizobium shewense ERR11T (JQ810006.1)
Bradyrhizobium nitroreducens TSA1T (NZ_LFJC00000000.1)
Bradyrhizobium kavangense 14-3T (KM378311.1)
Bradyrhizobium nanningense CCBAU 53390T (KC509323.1)
Bradyrhizobium arachidis CCBAU 051107T (JX437682.1)
Bradyrhizobium zhanjiangense CCBAU 51778T (KC509312.1)
Bradyrhizobium frederickii CNPSo 3426T (MK682699.1)
Bradyrhizobium diversitatis CNPSo 4019T (JACEGD000000000)
Bradyrhizobium liaoningense LMG 18230T (FM253266)
Bradyrhizobium subterraneum 58-2-1T (KM378349.1)
Bradyrhizobium yuanmingense CCBAU 10071T (FM253269)
Bradyrhizobium agreste CNPSo 4010T (JACCHP000000000)
Bradyrhizobium glycinis CNPSo 4016T (JACCHQ000000000)
Bradyrhizobium daqingense CGMCC 1.10947T (JX437676.1)
Bradyrhizobium guangxiense CCBAU 53363T (KC509328.1)
Bradyrhizobium vignae 7-2T (KM378308.1)
Bradyrhizobium cajani AMBPC 1010T (NZ_WQNE01000005.1)
Bradyrhizobium ingae BR 10250T (KF927073.1)
Bradyrhizobium iriomotense EK05T (HQ587646.1)
Bradyrhizobium cytisi CTAW11T (JN186288.1)
Bradyrhizobium centrolobii BR 10245T (KF983827.3)
Bradyrhizobium neotropicale BR 10247T (KF983829.1)
Bradyrhizobium denitrificans LMG 8443T (FM253282.1)
Bradyrhizobium oligotrophicum LMG 10732T (KF962713.1)
Bradyrhizobium namibiense 5-10T (KM378306.1)
Bradyrhizobium jicamae PAC 68T (HQ587647)
Bradyrhizobium paxllaeri LMTR 21T (KP308154.1)
Bradyrhizobium lablabi CCBAU 23086T (JX437677)
Bradyrhizobium australiense WSM 1791 T (JAAVLX000000000)
Bradyrhizobium retamae Ro19T (KF962714.1)
Bradyrhizobium icense LMTR 13T (NZ_CP016428.1)
Bradyrhizobium murdochi WSM 1741T (AXAU00000000)
Bradyrhizobium archetypum WSM 1744T (JAAVLW000000000)
Bradyrhizobium erythrophlei CCBAU 53325T (MG811654.1)
Bradyrhizobium ivorense CI-1BT (KX388393.1)
Bradyrhizobium uaiense UFLA 03-164T (NZ_VKHP00000000)
Bradyrhizobium macuxiense BR 10303T (KX527969.1)
Bradyrhizobium embrapense CNPSo 2833T (HQ634910.1)
Bradyrhizobium elkanii USDA 76T (AM295348)
Bradyrhizobium ferriligni CCBAU 51502T (MG811655.1)
Bradyrhizobium brasilense UFLA 03-321T (KF452879.1)
Bradyrhizobium pachyrhizi PAC 48T (HQ587648.1)
Bradyrhizobium viridifuturi SEMIA 690T (KU724169.1)
Bradyrhizobium ripae WR4T (MF593098.1)
Bradyrhizobium mercantei SEMIA 6399T (NZ_MKFI01000000.1)
Bradyrhizobium tropiciagri CNPSo 1112T (HQ634909.1)
Xanthobacter autotrophicus Py2 (NC_009720)
83
91
82
96
91
72
80
97
91
99
99
92
86
95
78
0.020

Fig. S6

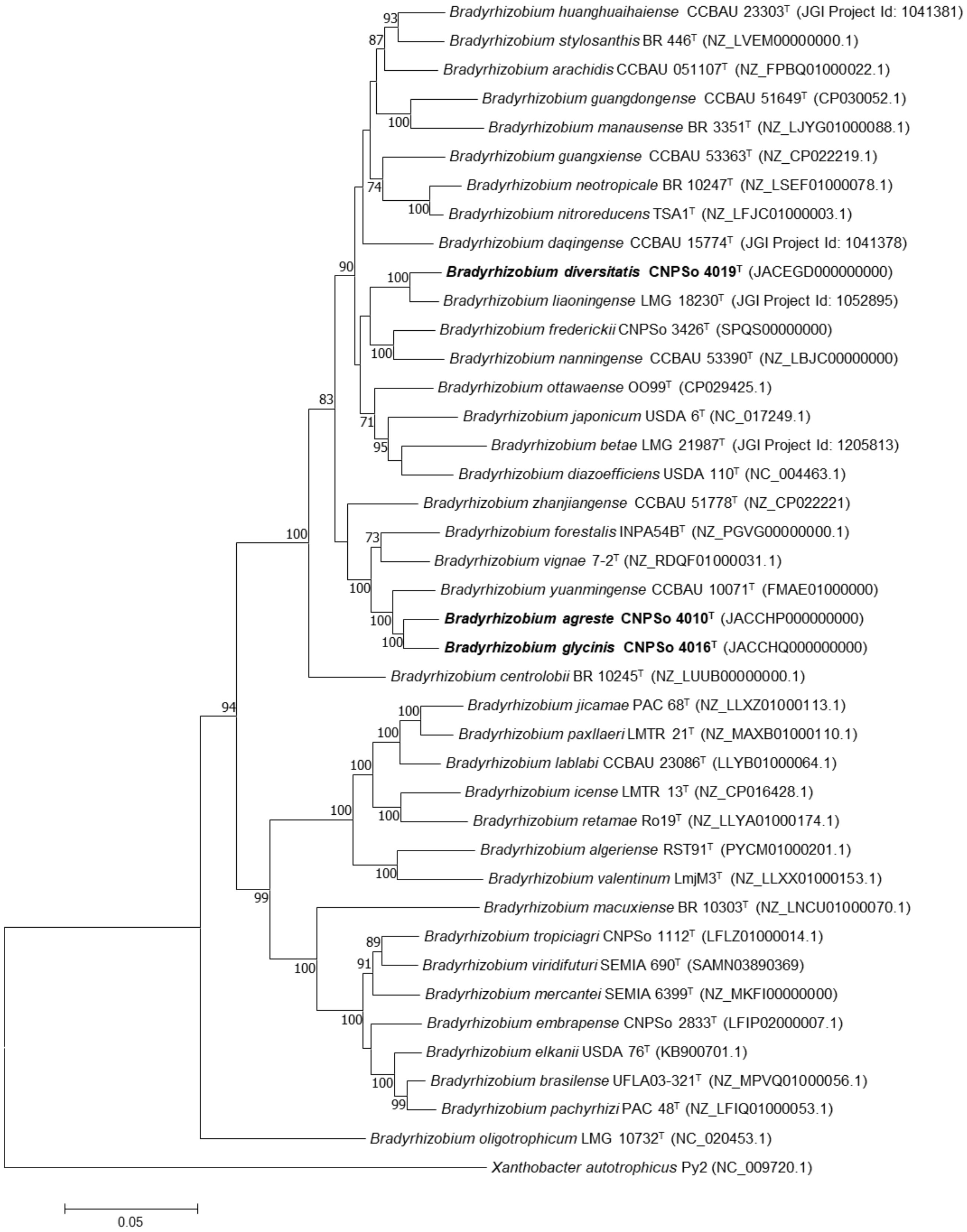
Bradyrhizobium huanghuaihaiense CCBAU 23303T (JGI Project Id: 1041381)
Bradyrhizobium stylosanthis BR 446T (NZ_LVEM00000000.1)
Bradyrhizobium arachidis CCBAU 051107T (NZ_FPBQ01000022.1)
Bradyrhizobium guangdongense CCBAU 51649T (CP030052.1)
Bradyrhizobium manausense BR 3351T (NZ_LJYG01000088.1)
Bradyrhizobium guangxiense CCBAU 53363T (NZ_CP022219.1)
Bradyrhizobium neotropicale BR 10247T (NZ_LSEF01000078.1)
Bradyrhizobium nitroreducens TSA1T (NZ_LFJC01000003.1)
Bradyrhizobium daqingense CCBAU 15774T (JGI Project Id: 1041378)
Bradyrhizobium diversitatis CNPSo 4019T (JACEGD000000000)
Bradyrhizobium liaoningense LMG 18230T (JGI Project Id: 1052895)
Bradyrhizobium frederickii CNPSo 3426T (SPQS00000000)
Bradyrhizobium nanningense CCBAU 53390T (NZ_LBJC00000000)
Bradyrhizobium ottawaense OO99T (CP029425.1)
Bradyrhizobium japonicum USDA 6T (NC_017249.1)
Bradyrhizobium betae LMG 21987T (JGI Project Id: 1205813)
Bradyrhizobium diazoefficiens USDA 110T (NC_004463.1)
Bradyrhizobium zhanjiangense CCBAU 51778T (NZ_CP022221)
Bradyrhizobium forestalis INPA54BT (NZ_PGVG00000000.1)
Bradyrhizobium vignae 7-2T (NZ_RDQF01000031.1)
Bradyrhizobium yuanmingense CCBAU 10071T (FMAE01000000)
Bradyrhizobium agreste CNPSo 4010T (JACCHP000000000)
Bradyrhizobium glycinis CNPSo 4016T (JACCHQ000000000)
Bradyrhizobium centrolobii BR 10245T (NZ_LUUB00000000.1)
Bradyrhizobium jicamae PAC 68T (NZ_LLXZ01000113.1)
Bradyrhizobium paxllaeri LMTR 21T (NZ_MAXB01000110.1)
Bradyrhizobium lablabi CCBAU 23086T (LLYB01000064.1)
Bradyrhizobium icense LMTR 13T (NZ_CP016428.1)
Bradyrhizobium retamae Ro19T (NZ_LLYA01000174.1)
Bradyrhizobium algeriense RST91T (PYCM01000201.1)
Bradyrhizobium valentinum LmjM3T (NZ_LLXX01000153.1)
Bradyrhizobium macuxiense BR 10303T (NZ_LNCU01000070.1)
Bradyrhizobium tropiciagri CNPSo 1112T (LFLZ01000014.1)
Bradyrhizobium viridifuturi SEMIA 690T (SAMN03890369)
Bradyrhizobium mercantei SEMIA 6399T (NZ_MKFI00000000)
Bradyrhizobium embrapense CNPSo 2833T (LFIP02000007.1)
Bradyrhizobium elkanii USDA 76T (KB900701.1)
Bradyrhizobium brasilense UFLA03-321T (NZ_MPVQ01000056.1)
Bradyrhizobium pachyrhizi PAC 48T (NZ_LFIQ01000053.1)
Bradyrhizobium oligotrophicum LMG 10732T (NC_020453.1)
Xanthobacter autotrophicus Py2 (NC_009720.1)
93
87
100
74
100
90
100
100
83
71
95
100
73
100
100
100
94
100
100
100
100
100
100
99
89
91
100
100
100
99
0.05

Fig. S7

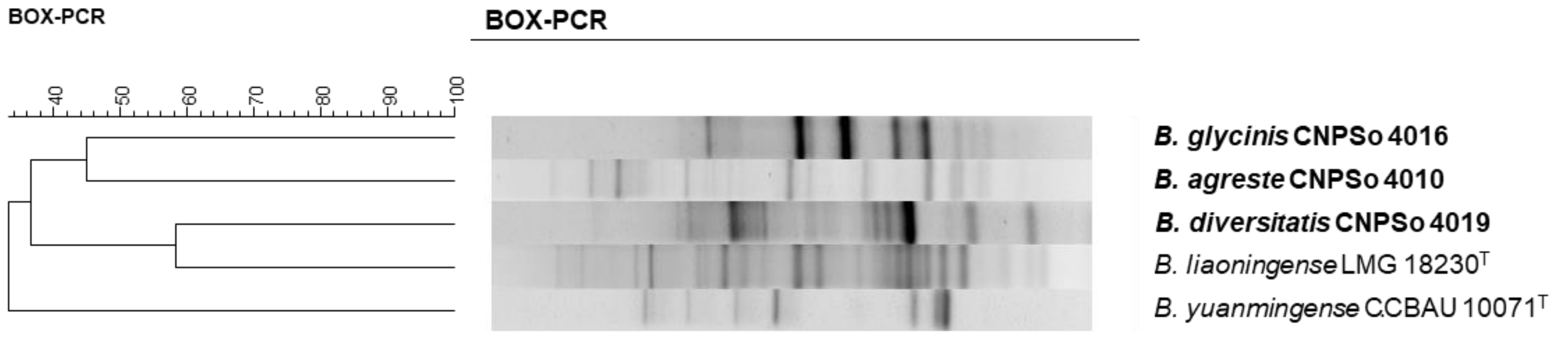

BOX-PCR
BOX-PCR
40
50
60
70
80
90
100
B. glycinis CNPSo 4016
B. agreste CNPSo 4010
B. diversitatis CNPSo 4019
B. liaoningense LMG 18230T
B. yuanmingense CCBAU 10071T

Fig. S8

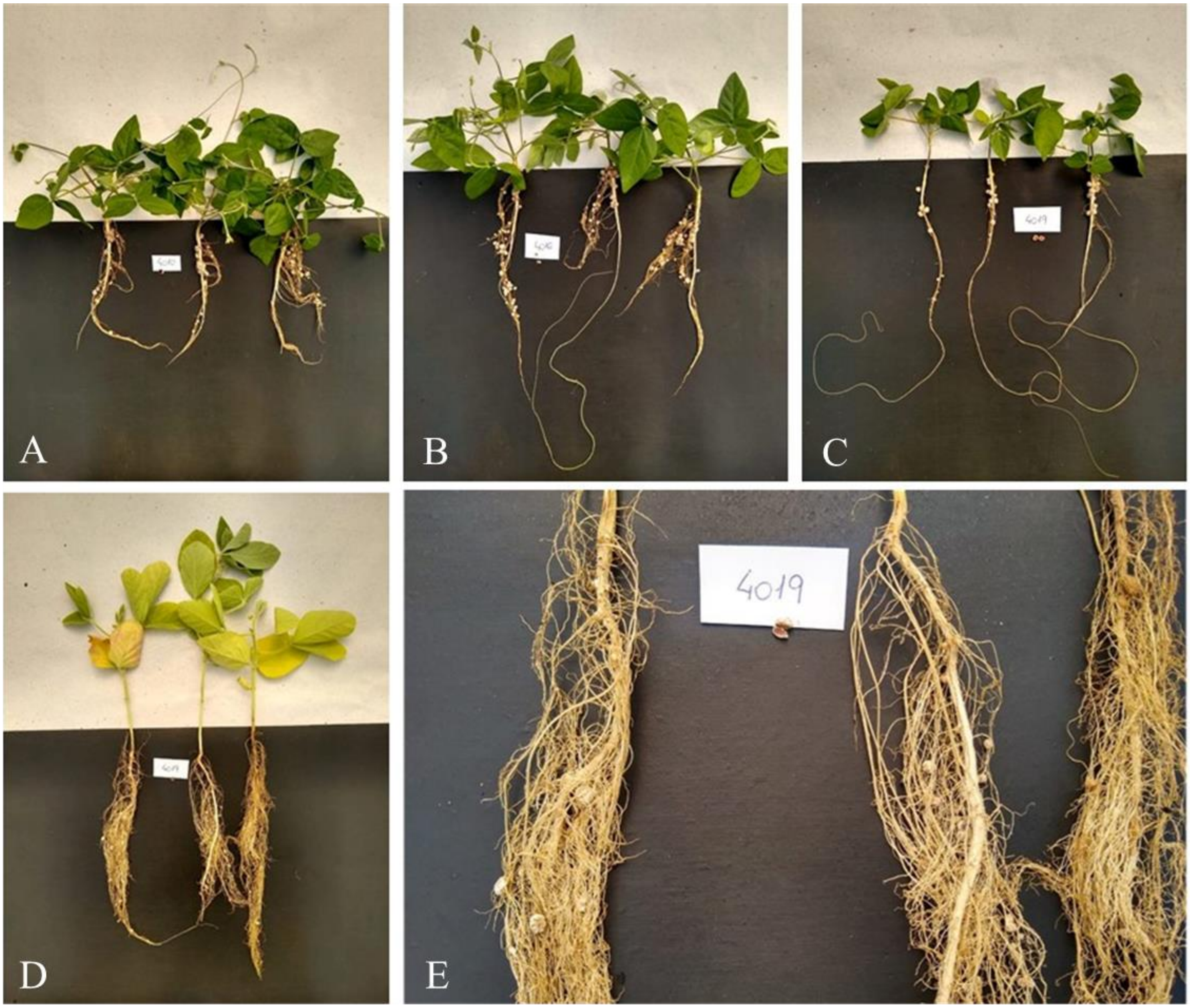

Fig. S9
